# Phylogenetic and biogeographic implications inferred by mitochondrial intergenic region analyses and ITS1-5.8S-ITS2 of the entomopathogenic fungi *Beauveria bassiana *and *B. brongniartii*

**DOI:** 10.1186/1471-2180-10-174

**Published:** 2010-06-16

**Authors:** Dimitri V Ghikas, Vassili N Kouvelis, Milton A Typas

**Affiliations:** 1 Department of Genetics, Faculty of Biology, University of Athens, Panepistimiopolis 15701, Athens, Greece

## Abstract

**Background:**

The entomopathogenic fungi of the genus *Beauveria *are cosmopolitan with a variety of different insect hosts. The two most important species, *B. bassiana *and *B. brongniartii*, have already been used as biological control agents of pests in agriculture and as models for the study of insect host - pathogen interactions. Mitochondrial (mt) genomes, due to their properties to evolve faster than the nuclear DNA, to contain introns and mobile elements and to exhibit extended polymorphisms, are ideal tools to examine genetic diversity within fungal populations and genetically identify a species or a particular isolate. Moreover, mt intergenic region can provide valuable phylogenetic information to study the biogeography of the fungus.

**Results:**

The complete mt genomes of *B. bassiana *(32,263 bp) and *B. brongniartii *(33,920 bp) were fully analysed. Apart from a typical gene content and organization, the *Beauveria *mt genomes contained several introns and had longer intergenic regions when compared with their close relatives. The phylogenetic diversity of a population of 84 *Beauveria *strains -mainly *B. bassiana *(n = 76) - isolated from temperate, sub-tropical and tropical habitats was examined by analyzing the nucleotide sequences of two mt intergenic regions (*atp*6-*rns *and *nad*3-*atp*9) and the nuclear ITS1-5.8S-ITS2 domain. Mt sequences allowed better differentiation of strains than the ITS region. Based on mt and the concatenated dataset of all genes, the *B. bassiana *strains were placed into two main clades: (a) the *B. bassiana s. l*. and (b) the "pseudobassiana". The combination of molecular phylogeny with criteria of geographic and climatic origin showed for the first time in entomopathogenic fungi, that the *B. bassiana s. l*. can be subdivided into seven clusters with common climate characteristics.

**Conclusions:**

This study indicates that mt genomes and in particular intergenic regions provide molecular phylogeny tools that combined with criteria of geographic and climatic origin can subdivide the *B. bassiana s.l. *entomopathogenic fungi into seven clusters with common climate characteristics.

## Background

*Beauveria *Vuill. is a globally distributed genus of soil-borne entomopathogenic hyphomycetes that is preferred as a model system for the study of entomopathogenesis and the biological control of pest insects [1]. The most abundant species of the genus is *Beauveria bassiana*, found in a wide host range of nearly 750 insect species, with extended studies on host-pathogen interactions at the molecular level and all the prerequisite knowledge for its commercial production [2]. *B. brongniartii*, the second most common species of the genus, has narrow host specificity and is well-studied as the pathogen of the European cockchafer (*Melolontha melolontha*), a pest in permanent grasslands and orchards [3]. Strains of both fungal species have been exploited as biological control agents (BCAs) [4,5].

As is usually the case for most mitosporic fungi, morphological characters are inadequate for delimiting species within a genus and this creates a continuing demand of screening for additional taxonomic characters. Consequently, through the years, several efforts have been made to genetically characterize or differentiate *Beauveria *species and strains, using various tools, including isozyme markers [6], karyotyping [7], vegetative compatibility groups [8], RAPD markers [9,10], rRNA gene sequencing and intron analyses [11,12], RFLPs and AFLPs [13-15], subtilisin protease genes [16], microsatellites [17,18] and combinations of rRNA gene complex and other nuclear genes [1,19,20]. These approaches provided valuable information on polymorphisms in populations of *B. bassiana*, with ITS sequences combined with other nuclear gene sequences being more reliable in taxonomic and phylogenetic studies [1,20,21]. Consequently, earlier assumptions that *Beauveria *is strictly asexual have been severely hampered by the recent discoveries of *Cordyceps *teleomorphs associated with *Beauveria *[1,22,23]. Thus, the extent to which the entire *Beauveria *genus is correlated with sexual *Cordyceps *remains to be examined and proved [1].

Mitochondrial DNA (mtDNA), due to its properties to evolve faster than the nuclear DNA, to contain introns and mobile elements and to exhibit extensive polymorphisms, has been increasingly used to examine genetic diversity within fungal populations [24-26]. In other mitosporic entomopathogenic fungi, such as *Metarhizium *[27], *Lecanicillium *[28] and *Nomurea *[29], mtDNA data compared favourably to data based on ITS combined with a single nuclear gene, for applications in phylogeny, taxonomy and species or strain -identification. In *Beauveria*, the use of mtDNA RFLPs or partial mtDNA sequences suggested that mtDNA can be equally useful for such studies [2,30].

In recent years, molecular techniques have revolutionized taxonomical studies and have provided strong evidence that some morphologically defined species consist of a number of cryptic species that are independent lineages with restricted distributions, for example, *Metarhizium anisopliae *[31], *Neurospora crassa *[32], and *Pleurotus cystidiosus *[33]. This has urged mycologists to extend their studies on large samples of individuals throughout the world, in order to establish robust phylogenies from the congruence of genealogies based on appropriately polymorphic gene sequences and to test hypotheses regarding the processes responsible for distribution patterns. Thus, the notion of phylogenetic species recognition and phylogeography was introduced as a powerful method for answering questions about distribution in an evolutionary context [34-36]. Phylogeography or phylogenetic biogeography emerge as the field that aims to understand the processes shaping geographic distributions of lineages using genealogies of populations and genes [37]. It is therefore, particularly important for genera like *Beauveria *for which only a few studies exist on strain variability and their geographic distribution and phylogenetic origins [6,13,16,17,20].

This work was undertaken to serve a dual purpose. Firstly, to further assess the usefulness of mtDNA sequences as species diagnostic tool, alone or in combination with the more commonly studied rRNA gene sequences (ITS), and secondly to infer relationships among a large population of *Beauveria *species and strains from different geographic origins, habitats and insect hosts. To achieve these targets we have analyzed the complete mt genomes of *B. bassiana *and *B. brongniartii*, selected the two most variable intergenic regions and constructed the phylogenetic relationships of a number of isolates for determining their biogeographic correlation.

## Results

### Gene content and genome organization

**Figure 1:**
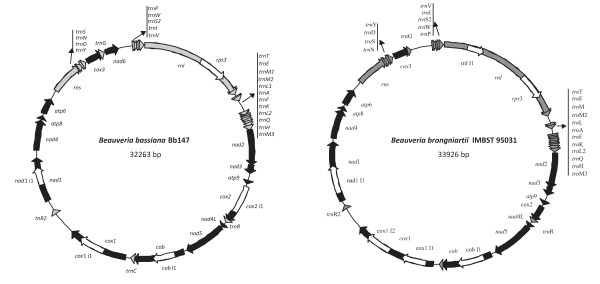
**Genetic organization of (a) *B. bassiana *strain Bb147 and (b) *B. brongniartii *strain IMBST 95031 mtDNA**. Protein-coding genes are marked with black arrows, and all other genes with gray arrows. Introns are shown with white arrows. Arrows indicate transcription orientation.

The mt genomes of the two *Beauveria *species had similar sizes, i.e., *B. brongniartii *IMBST 95031 33,926 bp and *B. bassiana *Bb147 32,263 bp, and both mapped circularly (Fig. 1). They contained all the expected genes found in typical mt genomes of ascomycetes (see Fig. 1; and Additional File 1, Table S1). Both genomes were compact and preserved the four synteny units proposed for Sordariomycetes, i.e., *rns*-*trn*_(1-5)_-*cox*3-*trn*_(1-5)_-*nad*6-*trn*_(2-9)_; *nad*1-*nad*4-*atp*8-*atp*6; *rnl*-*trn*_(11-12)_-*nad*2-*nad*3 and *nad*4L-*nad*5-*cob*-*cox*1 [38]. Important deduced differences in the gene content of the two genomes were found only when the intron number and insertion sites were included. This was also the case for mtDNA genome sequence of another *B. bassiana *isolate (Bb13) from China, recently deposited in GenBank (EU371503; 29.96 kb). When compared with our Bb147 mtDNA genome sequence, the two genomes were identical in gene order and nucleotide sequence (98-100%), for most of their sequence (approx. 28.1 kb). The difference in size -approx. 2.3 kb- was due to the absence from Bb13 of two introns, located in *cox*1 and *nad*1 genes of Bb147. Minor sequence differences were mostly in the intergenic regions with a preference to AT-rich areas, and were to a large extent SNP transitions (A/G and C/T) or single nucleotide insertions or deletions. The remaining differences were due to small insertions or deletions of 5-6 bp. The largest deletion (15bp) and the lowest sequence homology (86%) were observed in the intergenic region *cox*1- *trn*R2 (see Fig. 1).

### Introns

*B. bassiana *Bb147 contained five and *B. brongniartii *six introns, contributing to their total mtDNA genome size by 20.3% and 24.7%, respectively. All introns were group-I members, located in *rnl*, *cob*, *cox*1, *cox*2 and *nad*1 (Fig. 1; for details on exact positions of insertion and type of intron sub-group see Additional File 1, Table S1). All introns contained ORFs, i.e., the Rps3 homolog within the *rnl *gene (Bb*rnl*I and Bbr*rnl*I2), putative GIY-YIG homing endonucleases (Bb*cob*I1, *cox*2I1 and *nad*1I1) and the LAGLI-DADG endonuclease (Bb*cox*1I1 and Bbr*cox*1I1). The insertion positions of these introns were found to be conserved (identical sequences for at least 10 bp upstream and downstream of the insertion) for all known fungal complete mt genomes examined (36 in total). The only exception was the *cox*2 intron which was rarely encountered in other fungi. Interestingly, the additional intron detected in *rnl *of the *B. brongniartii *IMBST 95031 mt genome (positions 806-2102 of NC_011194 and Additional File 1, Table S1), was inserted at site not encountered before among the other complete mt genomes, i.e., the stem formed in domain II of *rnl *'s secondary structure. The target insertion sequence for the intron was GATAAGGTTG↓TGTATGTCAA and its intronic ORF encoded for a GIY-YIG endonuclease which shared homology (57% identity at the amino acid level) with I-PcI endonuclease of *Podospora curvicolla *(Acc. No. CAB 72450.1).

### Intergenic regions

Both mt genomes contained 39 intergenic regions amounting for 5,985 bp in *B. bassiana *and 5,723 bp in *B. brongniartii*, and corresponding to 18.6% and 16.9% of their total mt genome, respectively. The A+T content was very similar for these regions in both mt genomes (~74.5%) and the largest intergenic region was located between *cox*1-*trn*R2 with sizes 1,314 bp for *B. bassiana *and 1,274 bp for *B. brongniartii*, respectively. Analysis of these particular regions revealed large unique putative ORFs (*orf*387 and *orf*368 for both genomes) with no significant similarity to any other ORFs in Genbank. Additionally, many direct repeats were also located in the same regions (maximum length 37 bp and 53 bp for *B. bassiana *and *B. brongniartii*, respectively). All other intergenic regions in the two mt genomes had approximately the same sizes but with reduced nucleotide identity (sometimes as low as 78%). Therefore, the potential usefulness of mt intergenic sequence variation for intra- and inter- species discrimination and phylogenetic studies of *Beauveria *was examined following an *in silico *analysis based on criteria of size, complexity and suitability (for designing primers) of all *Beauveria *mt intergenic regions. More specifically, smaller than 200 bp interenic regions were excluded due to the few informative characters they contained, whereas ideal regions were considered those with sizes between 200-800 bp because they can be easily cloned and/or obtained by PCR. Regions containing *trn *genes -due to their cloverleaf structures- and regions with dispersed repetitive elements were avoided because their structures make them unsuitable for designing primers for PCR amplification (for details of all intergenic regions see Additional File 1, Table S1). Thus, the most suitable intergenic regions following the above criteria for the population analyses were *nad*3-*atp*9 and *atp*6-*rns*.

### Population and phylogenetic studies based on ITS1-5.8S-ITS2 and intergenic mt region sequences

PCR amplicons for the ITS1-5.8S-ITS2 region showed little variation in size, being almost identical for all *B. bassiana *(480-482 bp) and *B. brongniartii *(478-481 bp) isolates, but with sizeable differences for the other *Beauveria *species (471-512 bp). On the contrary, the intergenic *nad*3-*atp*9 and *atp*6-*rns *amplicons exhibited a much greater variability in sizes even within *B. bassiana *isolates, ranging from 259-332 bp for the former and 283-483 bp for the latter (Additional File 2, Table S2 and Additional File 3, Table S3), thus providing excellent tools for species or species-group identification. For example, using high-resolution agarose electrophoresis (data not shown), *nad*3-*atp*9 *B. bassiana *amplicons can be easily differentiated from the other *Beauveria *species and at the same time can be grouped into Clades A and C according to their sizes and in congruence to the classification proposed earlier [1] (Additional File 3, Table S3). Variability for the other *Beauveria *species was even greater, ranging from 84-302 bp and 249-441 bp for the *nad*3-*atp*9 and *atp*6-*rns*, respectively. When analyzed, these differences were found to be mainly due to deletions and/or additions of 3-5 nucleotides for *nad*3-*atp*9, scattered throughout this region, and rarely due to single point mutations. The *atp*6-*rns *sequence differences were primarily due to a 4-bp repeat (GCTT) inserted in the corresponding sequence up to 13 times (e.g., R184-483bp), thus providing in many cases excellent tools for isolate identification.

**Figure 2:**
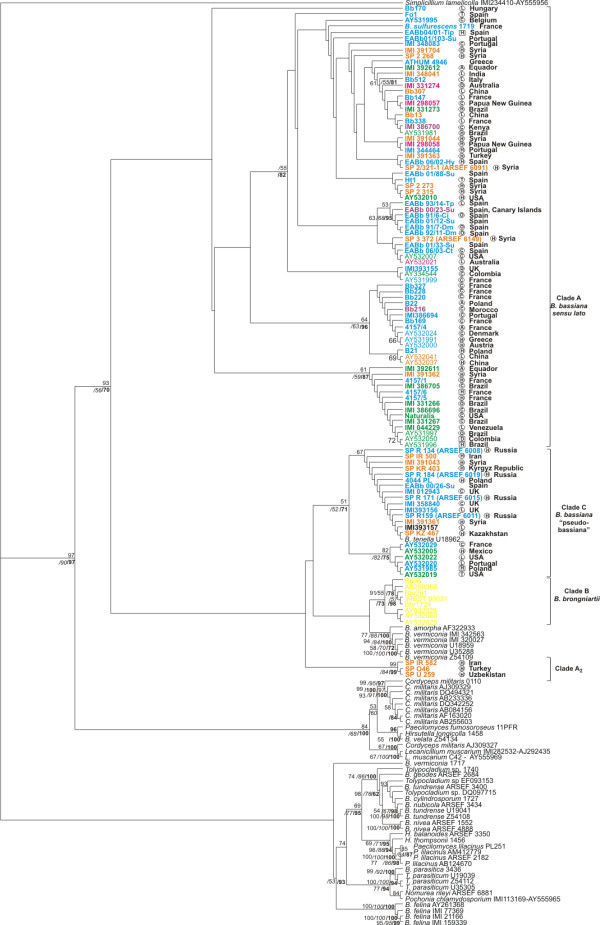
**Phylogenetic trees constructed from unambiguously aligned ITS1-5.8S-ITS2 domain, as produced by NJ analysis**. Clade credibility using NJ calculated from 1K replicates (upper numbers in roman), parsimony BS support calculated from 100 replicates (first lower numbers in italics) using PAUP and PPs produced by 2M generations (second lower numbers - in bold) using MrBayes, are shown. In the phylogenetics analysis of the ITS1-5.8S-ITS2 region, fungal species names and sequences obtained from GenBank are shown with their accession numbers in the figure. Fungal hosts are indicated as follows: in a circle, A, Araneida; C, Coleoptera; D, Diptera; H, Hemipetra; L, Lepidoptera; N, Nematoda; O, Orthoptera, T, Thysanoptera, R, Rotifera; ?, not known; in a square, H, Hymenoptera and no indication from soil or air. Geographic location is provided next to each isolate together with blue, orange, green, purple and magenta colour for the isolates originated from Europe, Asia, America, Africa and Oceania, respectively. *B. brongniartii *isolates are shown in yellow while isolates from all other Hypocreales species are provided in black.

**Figure 3:**
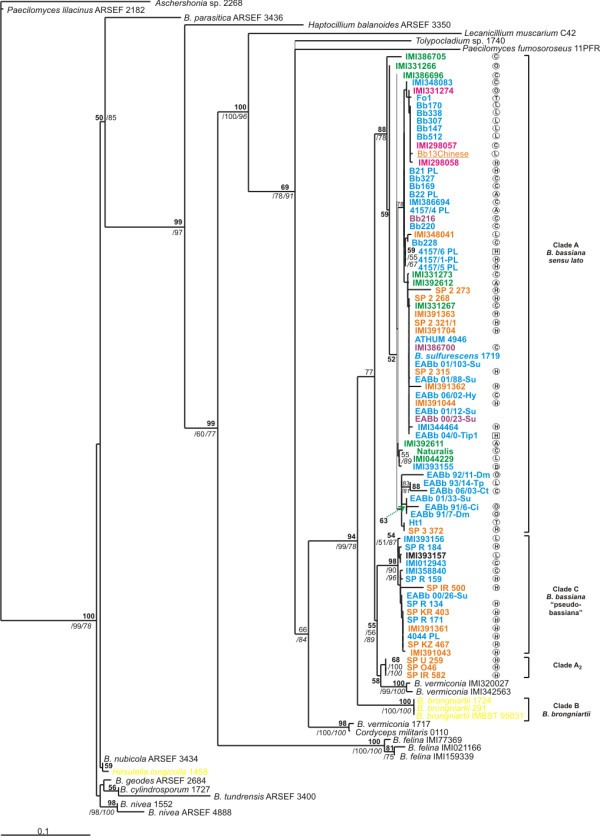
**Phylogenetic trees constructed from unambiguously aligned *nad*3-*atp*9 intergenic region, as produced by NJ analysis**. Clade credibility using NJ calculated from 1K replicates (upper numbers in roman), parsimony BS support calculated from 100 replicates (first lower numbers in italics) using PAUP and PPs produced by 1M generations (second lower numbers - in bold) using MrBayes, are shown. Fungal hosts, geographic locations and colour designations as in Fig. 2.

**Figure 4:**
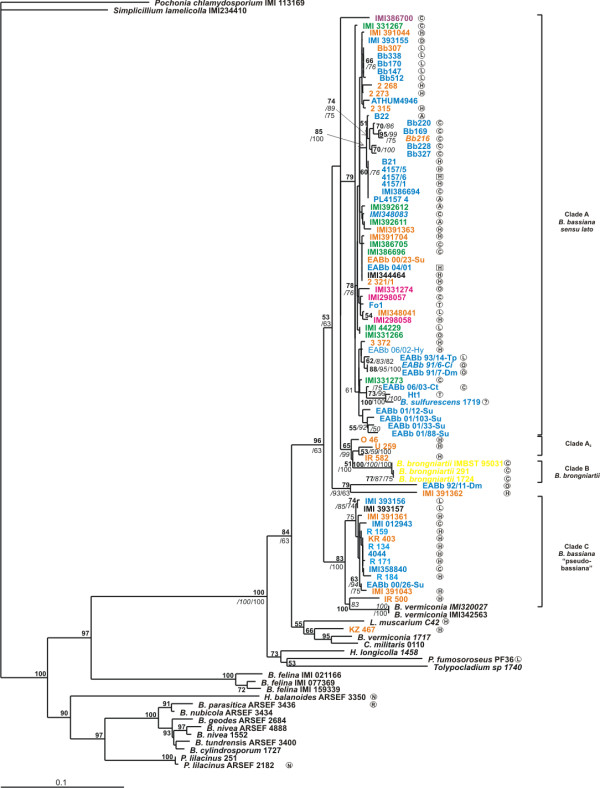
**Phylogenetic trees constructed from unambiguously aligned *atp6-rns *intergenic region, as produced by NJ analysis**. Clade credibility using NJ calculated from 1K replicates (upper numbers in roman), parsimony BS support calculated from 100 replicates (first lower numbers in italics) using PAUP and PPs produced by 1M generations (second lower numbers - in bold) using MrBayes, are shown. Fungal hosts, geographic locations and colour designations as in Fig. 2.

**Figure 5:**
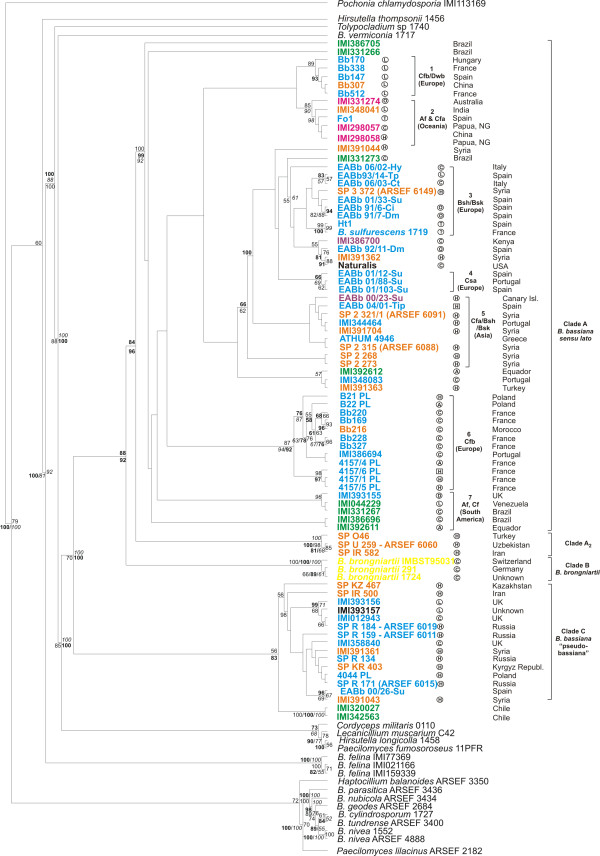
**Phylogenetic trees constructed from unambiguously aligned combined DNA sequences of the mt interegenic regions and the ITS domain as produced by NJ analysis**. Clade credibility using NJ calculated from 1K replicates (numbers in roman), parsimonial BS support calculated from 100 replicates (numbers in italics) using PAUP and PPs produced by 2M generations (numbers in bold) using MrBayes, are shown. Fungal hosts, geographic locations and colour designations as in Fig. 2. The 3 symbol Köppen-Geiger climate classification is also provided as follows: Af, Tropical Rain Forest; Am, Tropical Monsoon climate; Aw, Tropical wet and dry; BWh, Dry (arid and semiarid) desert low latitude climate; BWk, Dry (arid and semiarid) desert middle latitude climate; BSh, Dry (arid and semiarid) steppe low latitude climate; BSk, Dry (arid and semiarid) steppe middle latitude climate; Csa/Csb, Temperate Mediterranean climate; Cfa/Cwa, Temperate humid subtropical climate; Cfb/Cwb/Cfc, Temperate Maritime climate; Cwb, Temperate with dry winters climate; Cfc, Temperate Maritime Subarctic climate; Dfa/Dwa/Dsa, Hot summer Continental climate; Dfb/Dwb/Dsb, Warm summer Continental climate; Dfc/Dwc/Dsc, Continental Subarctic climate; Dfd/Dwd, Continental Subarctic climate with extremely severe winters [41].

Amplicon sequences from all isolates listed in Additional File 2, Table S2 were used to draw phylogenetic trees deduced from NJ analyses (Fig. 2, 3, 4 and 5), and parsimony and Bayesian methods were applied to examine the sensitivity of the resulting trees and tree topologies. Trees remained largely invariant to these manipulations and topologies were similar to a significant extent for each gene region tested independently of the phylogenetic method applied (symmetric difference values between the trees obtained with different methods for the same dataset are shown in Additional File 4, Table S4). Trees were rooted using as outgroups *Aschershonia *sp. and/or *Simplicillium lamelicolla *(both members of Hypocreales). Specifically, the phylogenetic tree produced from the ITS1-5.8S-ITS2 sequences obtained in this work and known related sequences from the databanks, divided the majority of *B. bassiana *strains into two major clades (Clade A and C), with marginal support of each clade (Fig. 2). The only exception was three strains (namely U259, O46 and IR582) that grouped together, at the base of the remaining *B. bassiana *strains with significant bootstrap (99 and 84% for the NJ and MP analyses, respectively) and Posterior Probability support (99% for the BI analysis). Similarly, the three *B. brongniartii *strains, grouped with the respective sequences obtained from GeneBank and produced a sister clade to *B. bassiana*, whereas the *B. vermiconia *and *B. amorpha *strains were basal to *B. bassiana *and *B. brongniartii *(Fig. 2). They all clearly clustered to a group different from the other species of the order Hypocreales, with significant NJ (97%) and MP (90%) bootstrap support. Based on 265 informative characters, 2,700 most parsimonious trees were constructed with tree length of 1,106 steps [Consistency Index (CI) = 0.56, Homoplasy Index (HI) = 0.44, Retention Index (RI) = 0.86, Rescaled Consistency Index (RC) = 0.48]. The relatively small number of informative characters may explain the marginal MP bootstrap and PP support. The remaining previously known *Beauveria *species (*B. geodes*, *B. nubicola*, *B. tundrense *and *B. parasiticum*) grouped well with other *Tolypocladium *species as expected according to known taxonomic criteria [39,40].

Both mt intergenic regions were more variable than the nuclear ITS1-5.8S-ITS2 for the *B. bassiana *strains. MP analyses were based on 232 and 343 informative characters and produced 7,700 most parsimonious trees with tree lengths 750 (CI = 0.71, HI = 0.29, RI = 0.87, RC = 0.62) and 1,085 steps (CI = 0.68, HI = 0.37, RI = 0.87, RC = 0.59) for the *nad*3-*atp*9 and *atp*6-*rns *regions, respectively. *B. bassiana *strains clustered into the same two groups (Clade A and C) and again the three isolates (SP IR582, SP O46 and SP U259) were placed as a separate group, as in the ITS1-5.8S-ITS2 trees (Fig. 3 and 4). Strains of *B. brongniartii *were basal to those of *B. bassiana *with a significant bootstrap and posterior probability support (94%, 99% and 78% for NJ, MP bootstrap and BI, respectively) in the *nad*3-*atp*9 analysis (Fig. 3), while in the *atp*6-*rns *tree they presented an identical topology to the ITS dataset, as a sister species to Clade A with a 100% support for all methods applied (Fig. 4). Here again, *Beauveria *species were clearly differentiated from other Hypocreales species, with significant support (Fig. 3 and 4). In addition, mt datasets provided better support of Clade C *B. bassiana *strains than their nuclear counterpart, i.e., NJ (98%) and MP (90%) bootstrap support for the *nad*3-*atp*9 dataset (Fig. 3), and 83% and 100%, respectively, for *atp*6-*rns *(Fig. 4). For both mt intergenic regions Clade C *B. bassiana *strains clustered as a sister group with the two *B. vermiconia *strains (i.e., IMI 320027 and IMI 342563), with the addition of the three independent *B. bassiana *isolates in the case of *nad*3-*atp*9.

In relation to insect host order, a "loose host-associated cluster" was observed only for Clade A strains, whereas Clade C *B. bassiana *strains were more diverse and no relation to host origin could be detected. Interestingly, the association of *B. bassiana *strain clusters with their insect host origin was more consistent with the *nad*3-*atp*9 data, than with data derived from *atp*6-*rns *analysis.

### Concatenated sequence analysis and evidence for host and climate associations of the clades

**Figure 6:**
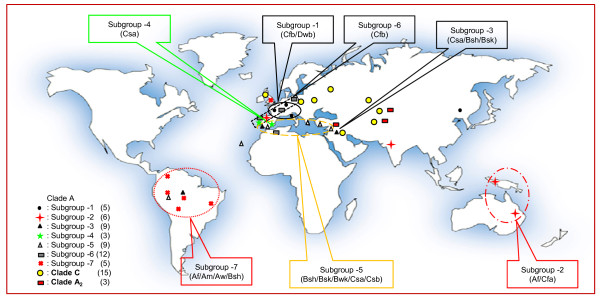
**Grouping of *B. bassiana sensu lato *strains (Clade Α) as well as Clade C and A**_**2**_**, according to their geographic distribution, climate conditions and molecular data (concatenated datasets from ITS1-5.8S-ITS2, *nad*3-*atp*9 and *atp*6-*rns*)**. The 3 symbol Köppen-Geiger climate classification is as shown in Fig. 5.

To fully integrate and exploit all the above information, a tree was constructed based on the concatenated ITS1-5.8S-ITS2, *atp*6-*rns *and *nad*3-*atp*9 sequences. Parsimony analysis provided more than 10,000 trees after exploiting 575 informative characters and the tree length was based on 1,895 steps (CI = 0.612, HI = 0.388, RI = 0.858, RC = 0.576). Analysis of the same dataset with NJ and BI methods produced similar trees with identical topologies wherever there was a strong support (Fig. 5). As in every tree produced by the analysis of a single gene region, *B. bassiana *strains grouped again into the same two major groups. The three isolates that were placed basally to the remaining *B. bassiana *remained independent, with significant bootstrap support (NJ: 99%, Fig. 5; see also DNA sequence percentage identity in comparisons of members of Clade A_2 _with members of Clades A and C in Additional File 5, Table S5). The most interesting feature of the concatenated data tree was that *B. bassiana *strains of Clade A could be divided further into seven distinct sub-groups that showed a "loose" association with their host (Fig. 5). This association was strengthened if the fungi were clustered according to their geographic and climatic origin (Fig. 6). More precisely, sub-groups 1, 3, 4 and 6 contained strains from Europe with five, nine, three and twelve members, respectively (Additional File 3, Table S3). Sub-group 1 strains were derived from France, Hungary and Spain (with a single strain from China). They were all isolated from a Lepidopteran host and originated from temperate maritime and continental microthermal climates (Cfb/Dwb) according to the Köppen-Geiger classification [41]. The three strains of sub-group 2 were isolated from Oceania (one from Australia and two from Papua New Guinea). To these, an Indian (Cfa), a Chinese (Cfa) and a Spanish (Csa) strain were also added, i.e., fungal strains from regions with temperate humid subtropic and Mediterranean climates, resembling the climate of the Oceanic Cfa [41]. Sub-groups 3 and 4 consisted almost exclusively of European strains (9 and 3, respectively) from regions with Mediterranean climate, such as Spain, Portugal and Italy. On the other hand, 12 strains from regions of Europe with maritime temperate climates (Cfb) formed a well-supported group (87 and 92% NJ and MP bootstrap and 94% PP support) presented as sub-group 6. All nine strains of sub-group 5 were from regions with dry arid, semiarid (BSh, BSk and BWk) and temperate (Csa and Csb) climates in Asia and Europe, while the South American (6) from tropic (Af, Am and Aw) and dry arid/semiarid (BSh) climates may be named as sub-group 7.

## Discussion

Fungal mt genome size shows high divergence among the Pezizomycotina, ranging from 100.3 Kb for *Podospora anserina *(NC_001329) to 24.5 Kb for the entomopathogen *Lecanicillium muscarium *(AF487277). *Beauveria *mt genomes sizes were similar to those of other fungi of the order Hypocreales, e.g., *Fusarium oxysporum *(34.5 Kb; AY945289) and *Hypocrea jecorina *(42.1 Kb; NC_003388), but they were significantly larger (~40%) than the mt genomes of the other two known entomopathogenic fungi of the order, i.e., *M. anisopliae *(24.7 kb) [27] and *L. muscarium *(24.5 kb) [42]. Since the *Beauveria *mtDNAs contained the same protein and rRNA coding genes -also identical in sizes- with all above mt genomes, their larger sizes can be attributed to more introns and to longer intergenic regions.

Compared to mt genomes of plants and animals, fungal mt genomes are significantly richer in group I and II introns [43]. Divergence in intron content is a common feature among mt genomes of Pezizomycotina. At one extreme is the mt genome of *P. anserina *which contains 41 introns [44] and at the other are several fungi that contain a single intron in the *rnl *genes of their mt genomes (i.e., *L. muscarium and M. anisopliae*). The recently released mt genome of another *B. bassiana *isolate (EU371503) also presented fewer introns than the genomes that we analyzed. These data support and extend previous evidence for intronic variability among strains of the same *Beauveria *species [14,16]. However, introns usually share common futures like insertion sites, encoded ORFs and total length [43,45]. Exceptions are noteworthy, not only because they suggest tools for the discrimination of the fungus but also because they provide information valuable to our understanding of fungal evolution [46-48]. In that respect, intron Bbr*rnl*1 inserted within domain II of *rnl*'s secondary structure was located in a novel (unique) site amongst the 36 Ascomycota complete mt genomes examined (Additional File 6, Table S6). Even though introns have been found in the same domain in Basidiomycota, for example *Agrocybe aegerita *[49], the uniqueness of this insertion site is of great importance to ascomycetes, as it may be a result of horizontal intron transfer. The fact that this intron encodes for a GIY-YIG homing endonuclease which shares homology with ORFs in introns located in different genes in other fungal genomes further strengthens the hypothesis of horizontal transfer. Yet, such a hypothesis remains to be experimentally tested.

Recently, a thorough attempt was made to determine associations of morphological characteristics with molecular data in *Beauveria *species [1]. Based on ITS1-5.8S-ITS2 and EF-1a sequences 86 exemplar isolates were examined and assigned to six major clades (A-F), where all known *Beauveria *species were included. *B. bassiana *isolates were grouped into two unrelated and morphologically indistinguishable clades (Clades A and C), while *B*. *brongniartii *formed a third sister clade to the other two (designated as Clade B). A new species, *B. malawiensis*, was later introduced and placed as sister clade to clade E [50], and several other *B. bassiana *isolates pathogenic to the coffee berry borer from Africa and the Neotropics were added to Clades A and C [22]. Our results from the ITS1-5.8S-ITS2 dataset are in full agreement with the grouping into Clades A-C and this division of *B. bassiana *isolates into two distinct clades is further supported by the mt intergenic region and the concatenated datasets with the best so far known bootstrap values. Mt genomes present different evolutionary rates compared to the nuclear [51] and topologies provided by one evolutionary pathway may not always indicate the correct relationships. As indicated by our findings, combining information from two independent heritages (nuclear and mt) may offer the possibility to resolve phylogenetic ambiguities. Thus, the two unrelated and morphologically indistinguishable *B. bassiana *clades proposed by Rehner and Buckley [1], i.e., the "*B. bassiana s.l.*", which contains the authentic *B. bassiana *(Clade A), and the "pseudobassiana" clade, which remains to be described (Clade C), are fully supported by our combined mt and nuclear data. Equally well supported by bootstrap is the placement of *B. brongniartii *strains as a sister clade to *B. bassiana*. The consistent clustering of the three *B. bassiana *isolates (our Clade A_2 _in Fig. 5 and Additional File 5, Table S5), which grouped basally to other *B. bassiana *with any datasets, indicates that this group is possibly a cryptic complex of *B. bassiana*. Experimental work with these and other similar isolates will be needed to substantiate this hypothesis.

A generally accepted notion that insect hosts are related to certain genotypes of entomopathogenic fungi has been tested in several occasions in the past for *B. bassiana *and *B. brongniartii*. However, only a few cases supported a host - fungal genotype specificity. For instance, associations have been reported between *B. brongniartii *and *Melolontha melolontha*, *M. hippocastani *or *Hoplochelus marginalis *[17,52]. A common *B. bassiana *genotype was detected in isolates from *Ostrinia nubilalis *[10] and from *Alphitobius diaperinus *[53]. More often, *B*. *bassiana *isolates collected from the same insect species were found to be genetically dissimilar [54,55] or showed cross-infectivity [56]. Similarly, fungal isolates derived from different insect species, families or orders clustered together [57]. Our results from the concatenated mt and nuclear gene datasets come to an agreement with the latter view, since molecular variability showed no general correlation between strains and host and/or geographic origin. This indicates that *B. bassiana *is a generalized insect pathogen, and is in agreement which its world-wide distribution, the vast variety of hosts from which it has been isolated and its entomopathogenic and/or endophytic characteristics [1,58]. It is only in rare occasions that a particular genotype, like Clade A sub-group 1 isolates (Fig. 6; Table 1), may be associated with a particular host (*Ostrinia nubilalis*). In the case of *B. brongniartii *and under the light of previous analyses of larger fungal populations [17,52], the association between fungal genotypes and a particular host seem to be stricter.

**Table 1:** Data from the phylogenetic analyses

	ITS1-5.8S-ITS2	*atp*6-*rns*	*nad*3-*atp*9	Concatenated
Total characters	640	687	496	1823
Constant characters	258	222	155	642
Variable characters	117	122	109	382
Informative characters	265	343	232	799

Tree length	1106	1085	750	2918
Consistency Index (CI)	0.56	0.68	0.71	0.64
Homoplasy Index (HI)	0.44	0.37	0.29	0.36
Retention Index (RI)	0.86	0.87	0.87	0.83
Rescaled Consistency Index (RC)	0.48	0.59	0.62	0.53
Parsimonious trees	2700	7700	7700	4100

Data obtained from the phylogenetic analyses of the nuclear ITS1-5.8S-ITS2 and the two mitochondrial intergenic regions *atp*6-*rns *and *nad*3-*atp*9 for all isolates examined in this study.

An increasing number of studies point towards a broad correlation of fungal isolates with their place of origin and/or habitats [e.g., [18,21,30,59,60]]. Obviously, the factors that can influence *B. bassiana *population structures are many and can include: climate conditions, the range of temperatures in which the various isolates can grow in nature, humidity levels, UV exposure, habitat, cropping system and soil properties [18,27,59,61]. In addition, collection strategies, numbers of isolates tested, gene loci used and molecular methods applied for population analyses can greatly contribute to the variability recorded. Thus, although the description of the effects of a single variable on the population of entomopathogenic fungi in a habitat can give significant and useful ecological and agronomical information, there may be relationships among the different variables that must be studied in detail to adequately understand the source of genetic variability in these fungi [59,61]. Therefore, to increase our potential to detect correlations between molecular markers and environmental variables, we incorporated climate conditions in our analyses, based on the most widely accepted classification system, the Köppen-Geiger climate classification [41]. This approach allowed fungal isolates that were otherwise outside of a particular cluster to be embodied in this cluster. Also, with few exceptions, strains isolated from distant geographic regions, which however shared similar climatic conditions, clustered together. If an explanation had to be proposed, the isolation by distance (allopatry) cannot be ruled out [22]. During the last decade molecular phylogenetic studies concerning fungal taxa which are considered to be widespread have resulted in the recognition of allopatric cryptic sibling species [33,62]. The suggestion that some morphologically defined species consist of a number of cryptic species that are independent lineages with restricted distributions [36], may explain the phylogeographic distribution of the three *B. bassiana *isolates designated in group A_2 _in this work. In other words, even though they are morphologically indistinguishable from the rest *B. bassiana *isolates, all three have the same host and are originated from Asia (i.e., Iran, Turkey and Uzbekistan) with similar climate (Bsk/Csa/Dsa).

It may be argued, and indeed it is the case, that the fungal isolates studied in this work are geographically "biased", since they are predominantly isolated from insects found in Europe (40) and Asia (19), and to a lesser extend from other places in North and South America, Africa and Oceania (16 isolates). However, even with this worldwide distribution of the isolates studied, continental drifts, geological barriers, host restrictions and human activities may contribute to long-distance dispersal and result to mixed sub-grouping classification. For instance, sub-group 2 (Fig. 6) contains the Oceanic isolates, one from India and one from Britain. While the "Indian" isolate may be considered as an evolutionary result of the opening of the Weddell Sea when eastern (including Australia, New Zealand and India) and western Gondwana (including Africa and Northern South America) separated [63], the "British" isolate may only be explained by accepting long-distance dispersal due to the human intervention as the most probable way. In similar studies where fungal distribution was caused by the breakup of Pangaea to New and Old World, like the *Pleurotus cystidiosus *group [33], *Schizophyllum *[64] and *Lentinula *[65], several exceptions to this pattern were observed in each study and they were usually explained by rare, but recent long - distance dispersal. Thus, gene flow among geographically distant populations of *B. bassiana *may be attributed to the long-distance dispersal of fungal spores through a variety of different direct or indirect means including wind, migratory insect vectors, rainfall, flooding and human traffic. On the other hand, the fact that several *B. bassiana *isolates belonging to different phylogenetic clades have been found in the same geographic location (e.g., Fig. 5, clades 3 and 4) may indicate a sympatric diversification. There appears to be no single morphological, physiological, host range, or genetic marker characteristic that can alone resolve molecular phylogenies in *B. bassiana*. Therefore, a strictly vicariant scenario may be not supported with these datasets and the occurrence of long - distance dispersal may be an alternate feasible scenario which renders the genus *Beauveria *cosmopolitan with several cryptic species, as already have been shown in other fungal taxa [66-68]. Nevertheless, in view of the ecological complexities of this entomopathogenic fungus, it is evident that terminal lineages can only be found if experiments are performed using more hierarchical parameters (climate, habitat, ecology and biogeography) in combination with multiple gene analyses that include data both from nuclear and mitochondrial genes.

## Conclusions

The complete mt genomes of *B. bassiana *and *B. brongniartii *analysed in this work had the typical gene content and organization found in other Ascomycetes of the order Hypocreales, but contained more introns and longer intergenic regions. The latter features can serve as tools for inter- and intra- species specific analysis within the genus *Beauveria*. Two mt intergenic regions (*nad*3-*atp*9 and *atp*6-*rns*) provided valuable sequence information and good support for the discrimination of *Beauveria *species and the division of 76 *B. bassiana *isolates into two groups, namely the *B. bassiana sensu lato *and the *B. bassiana *"pseudo-bassiana". These findings were in agreement with phylogenetic inferences based on ITS1-5.8S-ITS2 and demonstrated that mt sequences can be equally useful with the universally approved ITS1-5.8S-ITS2 for phylogenetic analysis. Further, mt sequence phylogenies constantly supported the formation of a third *B. bassiana *group, clearly differentiated from the rest, thus hinting for the presence of cryptic species within *B. bassiana*. Concatenated data sets of sequences from the three regions studied (i.e., the two mt and the nuclear ITS sequences) supported the above conclusions and often combined with criteria of isolate and geographic and climatic origins offered a better resolution of the *B. bassiana s.l. *strains and showed for the first time in entomopathogenic fungi, that *B. bassiana s.l. *strains can be subdivided into seven distinct sub-groups with common climate characteristics.

## Methods

### Strains, growth conditions, and DNA extraction

Seventy six strains of *Beauveria bassiana*, 3 of *B. brongniartii *and 14 strains of 9 other *Beauveria *species, together with one representative from each of 11 species belonging to the order Hypocreales were examined and are listed in Additional File 2, Table S2 (a fungal collection kept in the Department of Genetics and Biotechnology, Athens University, Greece). All fungal isolates were derived from single conidial spores grown on Potato Dextrose Agar (PDA) plates and all cultures were started from single spore isolations. Liquid cultures were in 250 ml flasks containing 50 ml of medium, inoculated with a spore suspension to reach 10^5^/ml final spore concentration, on an orbital shaker at 150 rev min^-1^, 25°C, for 3-4 days. Mycelia were removed by vacuum filtration, lyophilized for 2-4 days, and ground in liquid nitrogen using a mortar and pestle. Small quantities of ground mycelia (50-100 mg) were used for the extraction of DNA as described [69].

### Construction of libraries, PCR amplification and sequencing of the complete mt genomes

Isolation and digestion of nuclear and mtDNA from *B. bassiana *strain Bb147 and *B. brongiartii *strain IMBST 95031 were performed as previously described [69]. *Eco*RI and *Hind*III restricted fragments of CsCl-purified mtDNA were ligated into vector pBluescript KS+ (Stratagene, Cedar Creek, TX), analysed, subcloned and sequenced, thus covering over 78-80% of their complete mtDNA. The rest of the mtDNA and overlapping junctions were determined through sequence analysis of long-expand PCR amplicons. For this purpose, previously designed primers were used as follows: nad1B, cox3B, atp6A [42], cox2R, LSUER [27], LSUSF [38], and NMS1, NMS2 [70]. The primer pairs and respective amplicon sizes are shown in Additional File 7 (Additional File 7, Table S7). No sequence differences were observed between cloned fragments and PCR amplicons for the overlapping regions. PCR amplifications were performed with the proof-reading polymerase Herculase (Stratagene), in a PTC-200 Gradient Peltier Thermal Cycler (MJ Research, Waltham, MA), according to the manufacturer's instructions. PCR products were cloned in vector pDrive (QIAGEN, Hilden, Germany), subcloned as smaller fragments to pBluescript SKII and sequenced. Sequencing was performed with the Thermo Sequenase Primer Cycle Sequencing kit (Amersham Biosciences, Amersham, UK), and the reactions were analyzed at a LICOR 4200 IR^2 ^automated sequencer. All fragments were sequenced in both directions. DNA similarity searches were performed with Basic Local Alignment Search Tool (BLAST 2.2.14) [71]. The tRNAs were predicted by tRNAscan-SE 1.21 [72]. Intron identification and characterization utilized the intron prediction tool RNAweasel [73].

### Phylogenetic analysis

The ITS1-5.8S-ITS2 region of the nuclear rRNA gene-complex and two mtDNA intergenic regions, namely *nad*3-*atp*9 and *atp*6-*rns*, were examined in all isolates. DNA extracts from each isolate were used as templates for amplification with primers VLITS1 with VLITS2 for the ITS region [74], atp6F and rnsR for the *atp*6-*rns*, and nad3F with atp9R for the *nad*3-*atp*9 mt intergenic regions [27]. All reactions were performed with Herculase polymerase (Stratagene) in a PTC-200 (MJ Research) thermocycler according to the manufacturer's protocol, with a minor modification involving lower annealing temperature (45°C) for all three pairs. Sequencing was performed as above. DNA sequence alignments were made using CLUSTALW [75] with the multiple alignment parameters set to default and then edited by visual inspection (the matrix of the concatenated dataset and its partitions is provided in Additional File 8). Maximum parsimony (MP), Neighbor-Joining (NJ) and Bayesian inference (BI) analyses were employed in order to create the phylogenetic trees. The PAUP* program ver. 4.0b10 [76] was used for NJ (using the Kimura-2 parameter model) and MP analyses with 1,000 and 10,000 replicates, respectively, and with random addition of taxa and tree-bisection reconnection branch swapping [76]. Reliability of nodes was assessed using 1,000 and 100 bootstrap iterations for NJ and MP analyses, respectively. The BI was performed with MrBayes ver. 3.1 [77]. A gamma distribution model of site variation was used, calculated with PAML [78]. For ITS1-5.8S-ITS2, *nad*3-*atp*9, *atp*6-*rns *and concatenated data sets, alpha (a) was 0.529, 0.966, 1.311 and 0.693, respectively. Two independent MCMCMC searches were run for each data set using different random starting points (number of generations: 1,000,000 for all datasets except for the concatenated set, where 2 million generations were needed) with sampling every 100 generations. Convergence was checked visually by plotting likelihood scores vs. generation for the two runs [the first 25% samples from the cold chain (relburnin = yes and burninfrac = 0.25) were discarded]. Based on this analysis, the burn-in was set to 10,000, as this was found to be clearly sufficient for the likelihood and the model parameters to reach equilibrium. Distances between trees produced by the same dataset but different method were computed with the Symmetric Difference of Robinson and Foulds [79] as implemented in program Treedist of the PHYLIP v.3.69 package [80].

### Nucleotide sequence accession numbers

The complete sequence of *B. bassiana *strain Bb147 and *B. brongniartii *strain IMBST 95031 have been submitted to GenBank [GenBank: EU100742 and GenBank: NC_011194, respectively]. Also, nucleotide sequences for ITS and mtDNA intergenic regions were submitted to GenBank database [GenBank: FJ972917-FJ972972, GenBank: FJ973054-FJ973076 and GenBank: EU086417-EU086434 for the ITS region, GenBank: FJ972973-FJ973028, GenBank: FJ973077-FJ973101 and GenBank: EU086435-EU086455 for intergenic region *nad*3-*atp*9 and GenBank: FJ972862-FJ972916, GenBank: FJ973029-FJ973053 and GenBank: EU086396-EU086416 for the intergenic region *atp*6-*rns*].

## Authors' contributions

DVG contributed to design and performed the experiments and analysis of the complete mt genomes and helped in the population study. VNK contributed to design, performed experiments on the population study and the phylogenetic analyses. MAT designed research and supervised all the work. All authors contributed to the manuscript and approved the final version.

## Supplementary Material

Additional File 1 **Genetic content of the (a) *B. bassiana *Bb147 mt genome (EU100742) and (b) *B. brongniartii *IMBST 95031 mt genome (NC_011194)**. Click here for file

Additional File 2 **The strains used in this study, their hosts, geographical/climate origin**. Click here for file

Additional File 3 **PCR amplicon sizes (in nucleotides) of all *B. bassiana *isolates studied for the mt intergenic regions *nad*3-*atp*9 and *atp*6-*rns***. ITS1-5.8S-ITS2 amplicons are not shown because they were more or less identical (ranging from 480-482 nt for all strains). Click here for file

Additional File 4 **Values of symmetric difference between the phylogenetic trees produced from ITS1-5.8S-ITS2, *nad*3-*atp*9, *atp*6-*rns *and the concatenated dataset with NJ, BI and MP methods**. Click here for file

Additional File 5 **DNA sequence comparisons (% identity) of ITS1-5.8S-ITS2, *nad*3-*atp*9 and *atp*6-*rns *intergenic regions for representative isolates of *B. bassiana *Clades A, A**_**2**_**, C**. Isolates from Clade A and its subgroups, in green cells (and number in parentheses); isolates from Clade C and Clade A_2 _in yellow and blue cells, respectively. Click here for file

Additional File 6 **The complete mt genomes of fungi used in comparison with *Beauveria *mt genomes**. The complete mt genomes of fungi used in this study (all in red), their taxonomy, accession numbers, genome length, number of proteins and structural RNAs. All other presently known fungal complete mt genomes are shown in black. Click here for file

Additional File 7 **PCR primer pairs used for the amplification of the complete mt genomes of *B. bassiana *Bb 147 and *B. brongniartii *IMBST 95031 and approximate amplicon sizes in bp**. Click here for file

Additional File 8 **Matrix of concatenated dataset and genes/regions partitions used for the construction of the phylogenetic trees**. Click here for file

## References

[B1] Rehner SA Buckley EP A *Beauveria *phylogeny inferred from nuclear ITS and EF1-α sequences: evidence for cryptic diversification and links to *Cordyceps *teleomorphs Mycologia 2005 97 84 98 10.3852/mycologia.97.1.84 16389960

[B2] Uribe D Khachatourians GG Restriction fragment length polymorphisms of mitochondrial genome of the entomopathogenic fungus *Beauveria bassiana *reveals high intraspecific variation Mycol Res 2004 108 1070 1078 10.1017/S0953756204000760 15506018

[B3] Keller S Kessler P Schweizer C Distribution of insect pathogenic soil fungi in Switzerland with special reference to *Beauveria brongniartii *and *Metarhizium anisopliae* Biocontol 2003 48 307 319 10.1023/A:1023646207455

[B4] Butt TM Kempken F Use of entomogenous fungi for the control of insect pests The Mycota XI. Agricultural applications 2002 Berlin, Heidelberg Springer-Verlag 111 134

[B5] Strasser H Vey A Butt TM Are there any risks in using entomopathogenic fungi for pest control, with particular reference to the bioactive metabolites of *Metarhizium*, *Tolypocladium *and *Beauveria *species? Biocontrol Sci Technol 2000 10 717 735 10.1080/09583150020011690

[B6] St Leger RJ Allee LL May B Staples RC Roberts DW World-wide distribution of genetic variation among isolates of *Beauveria *spp Mycol Res 1992 96 1007 1015 10.1016/S0953-7562(09)80108-1

[B7] Viaud M Couteaudier Y Levis C Riba G Genome organization in *Beauveria bassiana *electrophoretic karyotype, gene mapping, and telomeric fingerprinting Fungal Genet Biol 1996 20 175 183 10.1006/fgbi.1996.0033

[B8] Couteaudier Y Viaud M New insights into population structure of *Beauveria bassiana *with regard to vegetative compatibility groups and telomeric restriction fragment length polymorphisms FEMS Microbiol Ecol 1997 22 175 182 10.1111/j.1574-6941.1997.tb00369.x

[B9] Bidochka MJ McDonald MA St Leger RJ Roberts DW Differentiation of species and strains of entomopathogenic fungi by random amplification of polymorphic DNA (RAPD) Curr Genet 1994 25 107 113 10.1007/BF00309534 8087878

[B10] Maurer P Couteaudier Y Girard PA Bridge PD Riba G Genetic diversity of *Beauveria bassiana *and relatedness to host insect range Mycol Res 1997 101 159 164 10.1017/S0953756296002213

[B11] Neuveglise C Brygoo Y Riba G 28S rDNA group-I introns: a powerful tool for identifying strains of *Beauveria brongniartii* Mol Ecol 1997 6 373 381 10.1046/j.1365-294X.1997.00202.x 9131812

[B12] Wang C Li Z Typas MA Butt TM Nuclear large subunit rDNA group I intron distribution in a population of *Beauveria bassiana *strains: phylogenetic implications Mycol Res 2003 107 1189 1200 10.1017/S0953756203008505 14635767

[B13] Aquino M de Muro Mehta S Moore D The use of amplified fragment length polymorphism for molecular analysis of *Beauveria bassiana *isolates from Kenya and other countries, and their correlation with host and geographical origin FEMS Microbiol Lett 2003 229 249 257 10.1016/S0378-1097(03)00841-3 14680707

[B14] Coates BS Hellmich RL Lewis LC Nuclear small subunit rRNA group I intron variation among *Beauveria *spp provide tools for strain identification and evidence of horizontal transfer Curr Genet 2002 41 414 424 10.1007/s00294-002-0317-8 12228811

[B15] Neuveglise C Brygoo Y Vercambre B Riba G Comparative analysis of molecular and biological characteristics of *Beauveria brongniartii *isolated from insects Mycol Res 1994 98 322 328 10.1016/S0953-7562(09)80460-7

[B16] Wang C Shah FA Patel N Li Z Butt TM Molecular investigation on strain genetic relatedness and population structure of *Beauveria bassiana* Environ Microbiol 2003 5 908 915 10.1046/j.1462-2920.2003.00485.x 14510844

[B17] Coates BS Hellmich RL Lewis LC Allelic variation of a *Beauveria bassiana *(Ascomycota: Hypocreales) minisatellite is independent of host range and geographic origin Genome 2002 45 125 132 10.1139/g01-132 11908654

[B18] Enkerli J Widmer F Gessler C Keller S Strain-specific microsatellite markers in the entomopathogenic fungus *Beauveria brongniartii* Mycol Res 2001 105 1079 1087 10.1016/S0953-7562(08)61970-X

[B19] Aquino de Muro M Elliott S Moore D Parker BL Skinner M Reid W El M Bouhssini Molecular characterisation *of Beauveria bassiana *isolates obtained from overwintering sites of Sunn Pest (*Eurygaster *and *Aelia *species) Mycol Res 2005 109 294 306 10.1017/S0953756204001832 15912946

[B20] Rehner SA Posada F Buckley EP Infante F Castillo A Vega FE Phylogenetic origins of African and Neotropical *Beauveria bassiana s. l. *pathogens of the coffee berry borer, *Hypothenemus hampei* J Invertebr Pathol 2006 93 11 21 10.1016/j.jip.2006.04.005 16806258

[B21] Meyling NV Lübeck M Buckley EP Eilenberg J Rehner SA Community composition, host range and genetic structure of the fungal entomopathogen *Beauveria *in adjoining agricultural and seminatural habitats Mol Evol 2009 18 1282 1293 10.1111/j.1365-294X.2009.04095.x 19226319

[B22] Li ZZ Li CR Huang B Fan MZ Discovery and demonstration of the teleomorph of *Beauveria bassiana *(Bals.) Vuill., an important entomogenous fungus Chinese Sci Bull 2001 46 751 753

[B23] Sung GH Hywel-Jones NL Sung JM Luangsa-ard JJ Shrestha B Spatafora JW Phylogenetic classification of Cordyceps and the clavicipitaceous fungi Studies Mycol 2007 57 5 59 10.3114/sim.2007.57.01 PMC2104736 18490993

[B24] Hegedus DD Khachatourians GG Identification of molecular variants in mitochondrial DNAs of members of the genera *Beauveria*, *Verticillium*, *Paecilomyces*, *Tolypocladium *and *Metarhizium* Appl Environm Microbiol 1993 59 4283 4288 10.1128/aem.59.12.4283-4288.1993 PMC195897 16349124

[B25] Mavridou A Typas MA Intraspecific polymorphism in *Metarhizium **anisopliae *var. *anisopliae *revealed by analysis of rRNA gene complex and mtDNA RFLPs Mycol Res 1998 102 1233 1241 10.1017/S0953756298006339

[B26] Sugimoto M Koike M Hiyama N Nagao H Genetic, morphological, and virulence characterization of the entomopathogenic fungus *Verticillium **lecanii* J Invertebr Pathol 2003 82 176 187 10.1016/S0022-2011(03)00014-4 12676554

[B27] Ghikas DV Kouvelis VN Typas MA The complete mitochondrial genome of the entomopathogenic fungus *Metarhizium **anisopliae *var. *anisopliae*: gene order and *trn *gene clusters reveal a common evolutionary course for all Sordariomycetes Arch Microbiol 2006 185 393 401 10.1007/s00203-006-0104-x 16552580

[B28] Kouvelis VN Sialakouma A Typas MA Mitochondrial gene sequences alone or combined with ITS region sequences provide firm molecular criteria for the classification of *Lecanicillium *species Mycol Res 2008 112 829 844 10.1016/j.mycres.2008.01.016 18501578

[B29] Sosa-Gomez DR Humber RA Hodge KT Binnek E Silva-Brandao KL Variability of the mitochondrial ssu rDNA of *Nomurea *species and other entomopathogenic fungi from Hypocreales Mycopathologia 2009 167 145 154 10.1007/s11046-008-9157-5 18830688

[B30] Kouvelis VN Ghikas DV Edgington S Typas MA Moore D Molecular characterization of isolates of *Beauveria **bassiana *obtained from overwintering and summer populations of Sunn Pest (*Eurygaster **integriceps*) Lett Appl Microbiol 2008 46 414 420 10.1111/j.1472-765X.2008.02331.x 18290810

[B31] Bidochka MJ Kamp AM Lavender TM Dekoning J JNA De Croos Habitat Association in Two Genetic Groups of the Insect-Pathogenic Fungus *Metarhizium anisopliae*: Uncovering Cryptic Species? Appl Environ Microbiol 2001 67 1335 1342 10.1128/AEM.67.3.1335-1342.2001 11229929 PMC92732

[B32] Dettman JR Jacobson DJ Taylor JW A multilocus genealogical approach to phylogenetic species recognition in the model eukaryote *Neurospora* Evolution 2003 57 2703 2720 14761051 10.1111/j.0014-3820.2003.tb01514.x

[B33] Zervakis G Moncalvo JM Vilgalys R Molecular phylogeny, biogeography and speciation in the mushroom species *Pleurotus cystidiosus *and allied taxa Microbiology 2004 150 715 726 10.1099/mic.0.26673-0 14993321

[B34] Avise JC Wollenberg K Phylogenetics and the origin of species Proc Natl Acad Sci USA 1997 94 7748 7755 10.1073/pnas.94.15.7748 9223259 PMC33696

[B35] Taylor JW Turner E Townsend JP Dettman JR Jacobson D Eukaryotic microbes, species recognition and the geographic limits of species: examples from the kingdom Fungi Phil Trans R Soc B 2006 361 1947 1963 10.1098/rstb.2006.1923 17062413 PMC1764934

[B36] Lumbsch HT Buchanan PK TW May Mueller GM Phylogeography and biogeography of fungi Mycol Res 2008 112 423 424 10.1016/j.mycres.2008.02.002 18346884

[B37] Avise JC Phylogeography: the history and formation of species 2000 Cambridge MA: Harvard University Press

[B38] Pantou MP Kouvelis VN Typas MA The complete mitochondrial genome of the vascular wilt fungus *Verticillium dahliae*: a novel gene order for *Verticillium *and a diagnostic tool for species identification Curr Genet 2006 50 125 136 10.1007/s00294-006-0079-9 16733756

[B39] von Arx JA *Tolypocladium*, a synonym of *Beauveria* Mycotaxon 1986 25 153 158

[B40] Index Fungorum http://www.indexfungorum.org/Names/Names.asp

[B41] Peel MC Finlayson BL McMahon TA Updated world map of the Köppen-Geiger climate classification Hydrol Earth Syst Sci 2007 11 1633 1644 10.5194/hess-11-1633-2007

[B42] Kouvelis VN Ghikas DV Typas MA The analysis of the complete mitochondrial genome of *Lecanicillium **muscarium *(synonym *Verticillium **lecanii*) suggests a minimum common gene organization in mtDNAs of Sordariomycetes: phylogenetic implications Fungal Genet Biol 2004 41 930 940 10.1016/j.fgb.2004.07.003 15341915

[B43] Lang BF Laforest MJ Burger G Mitochondrial introns: a critical view Trends Genet 2007 23 119 125 10.1016/j.tig.2007.01.006 17280737

[B44] Cummings DJ McNally KL Domenico JM Matsuura ET The complete DNA sequence of the mitochondrial genome of *Podospora anserina* Curr Genet 1990 17 375 402 10.1007/BF00334517 2357736

[B45] Clark-Walker GD Welstenholme DR, Jeon KW Evolution of mitochondrial genomes in fungi Mitochondrial Genomes 1992 San Diego, Academic Press 89 127

[B46] Pantou MP Kouvelis VN Typas MA The complete mitochondrial genome of *Fusarium oxysporum*: insights into fungal mitochondrial evolution Gene 2008 419 7 15 10.1016/j.gene.2008.04.009 18538510

[B47] Pramateftaki PV Kouvelis VN Lanaridis P Typas MA Complete mitochondrial genome sequence of the wine yeast *Candida zemplinina*: intraspecies distribution of a novel group-IIB1 intron with eubacterial affiliations FEMS Yeast Res 2008 8 311 327 10.1111/j.1567-1364.2007.00332.x 18081838

[B48] Zimmerly S Hausner G Wu XC Phylogenetic relationships among group II intron ORFs Nucleic Acids Res 2001 29 1238 1250 10.1093/nar/29.5.1238 11222775 PMC29734

[B49] Gonzalez P Barroso G Labarère J Molecular gene organisation and secondary structure of the mitochondrial large subunit ribosomal RNA from the cultivated Basidiomycota *Agrocybe aegerita*: a 13 kb gene possessing six unusual nucleotide extensions and eight introns Nucleic Acids Res 1999 27 1754 1761 10.1093/nar/27.7.1754 10076008 PMC148380

[B50] Rehner SA Aquino de Muro M Bischoff JF Description and phylogenetic placement of *Beauveria malawiensis *sp. nov. (Clavicipitaceae, Hypocreales) Mycotaxon 2006 98 137 145

[B51] Burger G Gray MW Lang BF Mitochondrial genomes: anything goes Trends Genet 2003 19 709 716 10.1016/j.tig.2003.10.012 14642752

[B52] Cravanzola F Piatti P Bridge PD Ozino OI Detection of genetic polymorphism by RAPD-PCR in strains of the entomopathogenic fungus *Beauveria **brongniartii *isolated from the European cockchafer (*Melolontha *spp.) Lett Appl Microbiol 1997 25 289 294 10.1046/j.1472-765X.1997.00226.x

[B53] Castrillo LA Wiegmann BM Brooks WM Genetic variation in *Beauveria bassiana *populations associated with the darkling beetle, *Alphitobius diaperinus* J Invertebr Pathol 1999 73 269 275 10.1006/jipa.1998.4835 10222180

[B54] Coates BS Hellmich RL Lewis LC *Beauveria bassiana *haplotype determination based on nuclear rDNA internal transcribed spacer PCR-RFLP Mycol Res 2002 106 40 50 10.1017/S0953756201005305

[B55] Urtz BE Rice WC RAPD-PCR characterization of *Beauveria bassiana *isolates from the rice water weevil *Lissorhoptrus oryzophilus* Lett Appl Microbiol 1997 25 405 409 10.1111/j.1472-765X.1997.tb00006.x

[B56] Glare TR Inwood AJ Morphological characterization of *Beauveria *spp. from New Zealand Mycol Res 1998 102 250 256 10.1017/S0953756297005005

[B57] Gaitan A Valderrama AM Saldarriaga G Velez P Bustillo A Genetic variability of *Beauveria bassiana *associated with the coffee berry borer *Hypothenemus hampei *and other insects Mycol Res 2002 106 1307 1314 10.1017/S0953756202006676

[B58] Quesada-Moraga E Landa BB Muñoz-Ledesma J Jiménez-Diáz RM Santiago-Alvarez C Endophytic colonization of opium poppy, *Papaver somniferum*, by an entomopathogenic *Beauveria bassiana *strain Mycopathologia 2006 161 323 329 10.1007/s11046-006-0014-0 16649082

[B59] Bidochka MJ Menzies FV Kamp AM Genetic groups of the insect-pathogenic fungus *Beauveria bassiana *are associated with habitat and thermal growth preferences Arch Microbiol 2002 178 531 537 10.1007/s00203-002-0490-7 12420176

[B60] Fernandes EKK Moraes AML Pacheco RS Rangel DEN Miller MP Bittencourt VREP Roberts DW Genetic diversity among Brazilian isolates of *Beauveria **bassiana*: comparisons with non-Brazilian isolates and other *Beauveria *species J Appl Microbiol 2009 107 760 774 10.1111/j.1365-2672.2009.04258.x 19486413

[B61] Quesada-Moraga E Navas-Cortés JA Maranhao EAA Ortiz-Urquiza A Santiago-Álvarez C Factors affecting the occurrence and distribution of entomopathogenic fungi in natural and cultivated soils Mycol Res 2007 111 947 966 10.1016/j.mycres.2007.06.006 17766099

[B62] Goodwin SB Legard DE Smart CD Levy M WE Fry Gene flow analysis of molecular markers confirms that *Phytophtora **mirabilis *and *P. infestens *are separate species Mycologia 1999 91 796 810 10.2307/3761533

[B63] McLoughlin S The breakup history of Gondwana and its impact of pre-Cenozoic floristic provincialism Aust J Bot 2001 49 271 300 10.1071/BT00023

[B64] James TY Moncalvo JM S Li Vilgalys R Polymorphism at the ribosomal DNA spacers and in its relation to breeding structure of the widespread mushroom *Schizophyllum commune* Genetics 2001 157 149 161 11139499 10.1093/genetics/157.1.149 PMC1461461

[B65] Hibbett DS Shiitake mushrooms and molecular clocks: historical biogeography of *Lentinula* J Biogeogr 2001 28 231 241 10.1046/j.1365-2699.2001.00528.x

[B66] Hosaka K Castellano MA Spatafora JW Biogeography of Hysterangiales (Phallomycetidae, Basidiomycota) Mycol Res 2008 112 448 462 10.1016/j.mycres.2007.06.004 18314317

[B67] Moncalvo JM Buchanan PK Molecular evidence for long distance dispersal across the Southern Hemisphere in the *Ganoderma applanatum-australe *species complex (Basidiomycota) Mycol Res 2008 112 425 436 10.1016/j.mycres.2007.12.001 18314318

[B68] Vizzini A Zotti M Mello A Alien fungal species distribution: the study case of *Favolaschia calocera* Biol Invasions 2009 11 417 429 10.1007/s10530-008-9259-5

[B69] Typas MA Griffen AM Bainbridge BW Heale JB Restriction fragment length polymorphisms in mitochondrial DNA and ribosomal RNA gene complexes as an aid to the characterization of species and sub-species populations in the genus *Verticillium* FEMS Microbiol Lett 1992 95 157 162 10.1111/j.1574-6968.1992.tb05359.x

[B70] White TJ Bruns TD Lee S Taylor J Innis MA, Gelfand DH, Sninsky JJ, White TJ Amplification and direct sequencing of fungal ribosomal RNA genes for phylogenetics PCR Protocols 1990 San Diego, Academic Press 315 322

[B71] Altschul SF Madden TL Schaffer AA Zhang J Zhang Z Miller W Lipman DJ Gapped BLAST and PSI-BLAST: a new generation of protein database search programs Nucleic Acids Res 1997 25 3389 3402 10.1093/nar/25.17.3389 9254694 PMC146917

[B72] Lowe TM Eddy SR tRNAscan-SE: a program for improved detection of transfer RNA genes in genomic sequence Nucleic Acids Res 1997 25 955 964 10.1093/nar/25.5.955 9023104 PMC146525

[B73] RNAweasel http://megasun.bch.umontreal.ca/RNAweasel

[B74] Pantou MP Strunnikova OK Shakhnazarova VY Vishnevskaya NA Papalouka VG Typas MA Molecular and immunochemical phylogeny of *Verticillium *species Mycol Res 2005 109 889 902 10.1017/S0953756205003345 16175791

[B75] Thompson JD Higgins DG Gibson TJ CLUSTAL W: improving the sensitivity of progressive multiple sequence alignment through sequence weighting, position specific gap penalties and weight matrix choice Nucleic Acids Res 1994 22 4673 4680 10.1093/nar/22.22.4673 7984417 PMC308517

[B76] Swofford DL PAUP: Phylogenetic Analysis Using Parsimony (* and other methods) 4.0 Beta 2002 Sunderland, MA, Sinauer

[B77] Ronquist FR Huelsenbeck JP MRBAYES 3: Bayesian phylogenetic inference under mixed models Bioinformatics 2003 19 1572 1574 10.1093/bioinformatics/btg180 12912839

[B78] Yang Z PAML: a program package for phylogenetic analysis by maximum likelihood Comput Appl Biosci 1997 13 555 556 9367129 10.1093/bioinformatics/13.5.555

[B79] Robinson DR Foulds LR Comparison of phylogenetic trees Math Biosci 1981 53 131 147 10.1016/0025-5564(81)90043-2

[B80] Felsenstein J PHYLIP (Phylogeny Inference Package) version 3.6 2005 Distributed by the author Department of Genome Sciences, University of Washington, Seattle

