# Supplementary material for: Phylogenetic and biogeographic implications inferred by mitochondrial intergenic region analyses and ITS1-5.8S-ITS2 of the entomopathogenic fungi Beauveria bassiana and B. brongniartii
Source: BMC Microbiol. 2010 Jun 16;10:174. doi: 10.1186/1471-2180-10-174 (PMC2896372; doi:10.1186/1471-2180-10-174)
Supplement: Additional File 1 — Genetic content of the (a) B. bassiana Bb147 mt genome (EU100742) and (b) B. brongniartii IMBST 95031 mt genome (NC_011194). [file 1471-2180-10-174-S1.DOC]

**Additional File 1, Table S1 - Genetic content of the (a) *B. bassiana* Bb147 mt genome (EU100742) and (b) *B. brongniartii*** IMBST 95031 mt genome (NC_011194).

| 1. **Gene** | **Position** | **Size (bp)** | **Gene** | **Position** | **Size (bp)** |
| --- | --- | --- | --- | --- | --- |
| *rnl* | 20-5061 | 5042 | Intronic ORF (GIY-YIG) | 14655-15575 | 921 |
| Group ΙA intron | 2550-4376 | 1827 | *trnC* (Cys)-TGC | 16713-16782 | 70 |
| *rps*3 | 2834-4174 | 1341 | *cox*1 | 17023-19883 | 2861 |
| *trn*T (Thr)-ACA | 5074-5144 | 71 | Group ΙB intron | 18091-19355 | 1265 |
| *trn*E (Glu)-GAA | 5361-5433 | 73 | Intronic ORF (LAGLI-DADG) *,** | 18091-19344 | 1254 |
| *trn*M1 (Met)-ATG | 5435-5505 | 71 | *trnR2*(Arg)-AGA | 21198-21268 | 71 |
| *trn*M2 (Met)-ATG | 6185-6257 | 73 | *nad*1 | 21769-23950 | 2168 |
| *trn*L1 (Leu)-TTA | 6261-6342 | 82 | Group Ι intron | 22399-23473 | 1075 |
| *trn*A (Ala)-GCA | 6346-6417 | 72 | intronic ORF (GIY-YIG)** | 22578-23447 | 870 |
| *trn*F (Phe)-TTC | 6419-6491 | 73 | *nad*4 | 24271-25767 | 1497 |
| trnK (Lys)-AAA | 6504-6576 | 73 | *atp*8 | 25839-25985 | 147 |
| *trn*L2 (Leu)-CTA | 6578-6661 | 84 | *atp*6 | 26093-26884 | 792 |
| *trn*Q (Gln)-CAA | 6665-6737 | 73 | *rns* | 27324-28888 | 1565 |
| *trn*H (His)-CAC | 6775-6847 | 73 | *trn*Y *(Tyr)-TAC* | 28926-29011 | 86 |
| *trn*M3 (Met) | 6905-6976 | 72 | *trn*D *(Asp)-GAC* | 29015-29087 | 73 |
| *nad*2 | 6984-8687 | 1704 | *trn*S (Ser)-AGC | 29164-29244 | 81 |
| *nad*3 | 8687-9106 | 420 | *trn*N (Asn)-AAC | 29250-29320 | 71 |
| *atp*9 | 9407-9631 | 225 | *cox*3 | 29383-30192 | 810 |
| *cox*2 | 9743-11608 | 1866 | *trn*G *(*Gly*)-GGA* | 30238-30308 | 71 |
| Group ΙB intron | 9971-11086 | 1116 | *nad*6 | 30362-31000 | 639 |
| Intronic ORF (GIY-YIG) * | 9971*-10852 | 882 | *trn*V (Val)-GTA | 31832-31904 | 73 |
| *trn*R (Arg)-CGT | 11687-11757 | 71 | *trn*I (Ile)-ATC | 31909-31980 | 72 |
| *nad*4L | 11793-12062 | 270 | *trn*S2 (Ser)-TCA | 32018-32102 | 85 |
| *nad*5 | 12062-14047 | 1986 | *trn*W (Trp)-TGA | 32104-32185 | 72 |
| *cob* | 14202-16617 | 2416 | *trn*P (Pro)-CCA | 32191-32262 | 72 |
| Group ΙB intron | 14595-15834 | 1240 |  |  |  |

*: ORF is in frame with the precedent exon

**: ORF with alternate initiation codon

| **(b) Gene** | **Position** | **Size (bp)** | **Gene** | **Position** | **Size (bp)** |
| --- | --- | --- | --- | --- | --- |
| *rnl* | 10-6449 | 6440 | *trn*C (Cys)-TGC | 17122-17191 | 79 |
| Group ΙC intron | 806-2102 | 1297 | *cox*1 | 17431-21525 | 4095 |
| intronic ORF (GIY-YIG) | 1177-1989 | 810 | Group ΙB intron | 17812-19009 | 1198 |
| Group ΙA intron | 3838-5763 | 1906 | intronic ORF (LAGLI-DADG) | 18034-18960 | 927 |
| *rps*3 | 4122-5537 | 1416 | Group ΙB intron | 19697-20976 | 1280 |
| *trn*T (Thr)-ACA | 6462-6532 | 71 | intronic ORF (GIY-YIG) *,** | 19697-20965 | 1269 |
| *trn*E (Glu)-GAA | 7047-7119 | 73 | *trn*R2(Arg)-AGA | 22800-22870 | 71 |
| *trn*M1 (Met)-ATG | 7121-7191 | 71 | *nad*1 | 23391-25901 | 2520 |
| *trn*M2 (Met)-ATG | 7573-7645 | 73 | Group ΙC intron | 23529-24932 | 1404 |
| *trn*L1 (Leu)-TTA | 7649-7730 | 82 | intronic ORF (GIY-YIG) ** | 23549-24583 | 1035 |
| *trn*A (Ala)-GCA | 7734-7805 | 72 | *nad*4 | 26207-27703 | 1497 |
| *trn*F (Phe)-TTC | 7807-7879 | 73 | *atp*8 | 27775-27921 | 147 |
| *trn*K (Lys)-AAA | 7892-7964 | 73 | *atp*6 | 28032-28823 | 792 |
| *trn*L2 (Leu)-CTA | 7966-8049 | 84 | *rns* | 29349-30930 | 1582 |
| *trn*Q (Gln)-CAA | 8053-8125 | 73 | *trn*Y *(Tyr)-TAC* | 30968-31053 | 86 |
| *trn*H (His)-CAC | 8168-8240 | 73 | *trn*D *(Asp)-GAC* | 31057-31129 | 73 |
| *trn*M3 (Met) | 8297-8368 | 72 | *trn*S (Ser)-AGC | 31200-31280 | 81 |
| *nad*2 | 8376-10082 | 1707 | *trn*N (Asn)-AAC | 31286-31356 | 71 |
| *nad*3 | 10082-10501 | 420 | *cox*3 | 31419-32228 | 810 |
| *atp*9 | 10791-11015 | 225 | *trn*G *(Gly)-GGA* | 32274-32344 | 71 |
| *cox2* | 11118-11867 | 750 | *nad*6 | 32398-33036 | 639 |
| *trnR* (Arg)-CGT | 11978-12048 | 71 | *trn*V (Val)-GTA | 33489-33561 | 73 |
| *nad*4L | 12085-12354 | 270 | *trn*I (Ile)-ATC | 33566-33637 | 72 |
| *nad*5 | 12354-14336 | 1983 | *trn*S2 (Ser)-TCA | 33675-33759 | 85 |
| *cob* | 14577-17050 | 2474 | *trn*W (Trp)-TGA | 33761-33832 | 72 |
| Group ΙD intron | 14970-16267 | 1298 | *trn*P (Pro)-CCA | 33844-33915 | 72 |
| intronic ORF (GIY-YIG) | 15143-15880 | 738 |  |  |  |

*: ORF is in frame with the precedent exon

**: ORF with alternate initiation codon
