# Supplementary material for: Phylogenetic and biogeographic implications inferred by mitochondrial intergenic region analyses and ITS1-5.8S-ITS2 of the entomopathogenic fungi Beauveria bassiana and B. brongniartii
Source: BMC Microbiol. 2010 Jun 16;10:174. doi: 10.1186/1471-2180-10-174 (PMC2896372; doi:10.1186/1471-2180-10-174)
Supplement: Additional File 2 — The strains used in this study, their hosts, geographical/climate origin. [file 1471-2180-10-174-S2.DOC]

## Additional File 2, Table S2 - The strains used in this study, their hosts, geographical/climate origin.

| **A/A** | **Strains*** | **Host** | **Origin-Climate**** |
| --- | --- | --- | --- |
|  | ***Beauveria bassiana*** |  |  |
| 1 | Bb147 | *Ostrinia nubilalis* (European corn borer) (Crambidae, Pyraloidea, Lepidoptera) | France, Centre – Cfb |
| 2 | Bb170 | *O. nubilalis* (Crambidae, Pyraloidea, Lepidoptera) | Hungary – Dwb |
| 3 | Bb307 | *O. nubilalis* (Crambidae, Pyraloidea, Lepidoptera) | China |
| 4 | Bb338 | *O. nubilalis* (Crambidae, Pyraloidea, Lepidoptera) | France, Haute Normandie – Cfb |
| 5 | Bb512 | *O. nubilalis* (Crambidae, Pyraloidea, Lepidoptera) | Italy |
| 6 | Fo1 | *Frankliniella occidentalis* (Thripidae, Thripoidea, Thysanoptera) | Spain |
| 7 | IMI 298057 | *Pantorhytes plutus* (Panthorhytes or Curculionidae, Curculionoidea, Coleoptera) | Papua Nea Guinea, Keravat – Af |
| 8 | IMI 298058 | *Pseudodoniella* *typica* (Miridae, Cimicoidea, Hemiptera) | Papua, Nea Guinea, Keravat – Af |
| 9 | IMI 331274 (ARSEF 356) | Grasshopper (Acrididae, Acridoidea, Orthoptera) | Australia, New South Wales, Armidale – Cfa |
| 10 | IMI 348041 | *Emmalocera depressella* (root borer) [Pyraloidea (Plotellidae, Lepidoptera)] | India, Haryana, Yamunanager – Cwa (Cfa) |
| 11 | IMI 044229 | *Diatrea saccharalis* (sugarcane borer) (Crambidae, Pyraloidea, Lepidoptera) | Venezuela – Am/Aw |
| 12 | IMI 331266 (ARSEF 1959) | unidentified insect (Acrididae, Acridoidea, Orthoptera) | Brazil, Bahia, Ribeira de Pombal – BSh |
| 13 | IMI 331267  (ARSEF 1480) | *Hypothenemus hampei* (coffee berry borer) (Curculionidae or **Scolytidae**, Antticidae, Coleoptera) | Brazil, Sao Paulo – Cfa (Cwa) |
| 14 | IMI 386696 | *Diabrotica* sp. (Chryssomelidae, Chrysomeloidea, Coleoptera) | Brazil (Amazonas) – Af |
| 15 | IMI 386705 | *Diabrotica speciosa* (Chryssomelidae, Chrysomeloidea, Coleoptera) | Brazil (Bahia) – BSh |
| 16 | IMI 392611 | (Araneidae, Araneoidea, Arachnida) | Equador – Af |
| 17 | IMI 393155 | *Phlebotomus papatasi* (Psychodidae, Psychodoidea, Diptera) | United Kingdom – Cfb |
| 18 | B21 PL | *Auchenorrhynchus* sp. (Heteroptera, Hemiptera) | Poland, Bialowieza National Park – Dfb |
| 19 | B22 PL | Spider (Araneidae, Araneoidea, Arachnida) | Poland, Siedlce – Dfb |
| 20 | Bb169  (ARSEF 1491) | *Sitona humeralis* (Curculionidae, Curculionoidea, Coleoptera) | France, Poitou – Cfb |
| 21 | Bb216 | *Sitona discoideus* (Curculionidae, Curculionoidea, Coleoptera) | Morocco – Csa/Bsk/Bsh |
| 22 | Bb220 | *Sitona discoideus* (Curculionidae, Curculionoidea, Coleoptera) | France, Centre – Cfb (Cwb) |
| 23 | Bb228 | Sitona discoideus (Curculionidae, Curculionoidea, Coleoptera) | France, Haute-Normandie – Cfb |
| 24 | Bb327 | *Sitona discoideus* (Curculionidae, Curculionoidea, Coleoptera) | France – Cfb |
| 25 | IMI 386694 | *Popillia japonica* (Scarabaeidae, Coleoptera) | Portugal – Csa/Csb |
| 26 | 4157/1 PL | *Anthocoris* sp. (Anthocoridae, Cimicoidea, Hemiptera) | France, Laon – Cfb |
| 27 | 4157/4 PL | small spider (Araneidae, Araneoidea, Arachnida) | France, Laon – Cfb (Cwb) |
| 28 | 4157/5 PL | *Anthocoris* sp. (Anthocoridae, Cimicoidea, Hemiptera) | France, Laon – Cfb |
| 29 | 4157/6 PL | Ichneumonidae, Hymenoptera | France, Laon – Cfb |
| 30 | ATHUM 4946 | Air-borne | Greece, Athens – Csa |
| 31 | EABb 00/23-Su | Soil, pasture | Canary Islands (Tenerife) – BWk |
| 32 | EABb 04/01-Tip | *Timaspis papaveris* (Cynipidae, Cynipoidea, Hymenoptera) | Spain, Seville (Carmona) – Csa |
| 33 | IMI 344464 | *Ctenarytaina eucalypti* (Psyllidae, Hemiptera) | Portugal, Torre Bela – Csb |
| 34 | IMI 348083 | *Phoracantha semipunctata* (Cerambycidae, Chrysomeloidea, Coleoptera) | Portugal, Tramagal, Caniceira – Csa |
| 35 | IMI 391044 | *E. integriceps* (Pentatomoidea, Hemiptera) | Syria – BSh/BSk/Csa |
| 36 | IMI 391363 | *E. integriceps* (Pentatomoidea, Hemiptera) | Turkey |
| 37 | IMI 391704 | *Eurygaster integriceps* (Pentatomoidea, Hemiptera) | Syria – BSh/BSk/Csa |
| 38 | IMI 392612 | spider adult (Araneidae, Araneoidea, Arachnida) | Equador – Af |
| 39 | SP 2 268 | *E. integriceps* (Pentatomoidea, Hemiptera) | Syria – BSh/BSk/Csa |
| 40 | SP 2 273 | *E. integriceps* (Pentatomoidea, Hemiptera) | Syria – BSh/BSk/Csa |
| 41 | SP 2 315 -  ARSEF 6088 | *E. integriceps* (Pentatomoidea, Hemiptera) | Syria, Al Kseabiia – BSh/BSk/Csa |
| 42 | SP 2 321/1 –  ARSEF 6091 | *E. integriceps* (Pentatomoidea, Hemiptera) | Syria, Al Kseabiia – BSh/BSk/Csa |
| 43 | EABb 01/12-Su | Soil non-cultivated area | Spain, Seville – Csa |
| 44 | EABb 01/88-Su | Soil, sunflower | Portugal, Villa Velha – Csa |
| 45 | EABb 01/103-Su | Soil, forest | Spain, Seville – Csa |
| 46 | EABb 01/33-Su | Soil, olive | Spain Cadiz – Csa |
| 47 | EABb06/02-Hy | *Hyalopterus pruni* (Aphididae, Aphidoidea, Hemiptera) | ? (Spain) |
| 48 | EABb 06/03-Ct | *Capnodis tenebrionis* (Buprestidae, Coleoptera) | ? (Spain) |
| 49 | EABb 91/6-Ci | *Calliptamus italicus* (Acrididae, Acridoidea, Orthoptera) | Spain |
| 50 | EABb 91/7-Dm | *Dociostaurus maroccanus* (Acrididae, Acridoidea, Orthoptera) | Spain"la Serena" Badajoz – BSk |
| 51 | EABb93/14-Tp | *Thaumetopoea pytiocampa* (Thaumetopoeidae, Noctuoidea) or Notodontidae, Lepidoptera | Spain, Cordoba forest – Csa |
| 52 | Ht1 | *Hoplothrips tritici* (Phlaeothripidae, Phlaeothripoidea, Thysanoptera) | Spain |
| 53 | IMI 331273 (ARSEF 757) | *Tenebrinoid* beetle (Tenebrionidae, Tenebrionoidea, Coleoptera) | Brazil, Ceara, Missaeo Velha– Aw/BSh |
| 54 | SP 3 372 -  ARSEF 6149 | *E. integriceps* (Pentatomoidea, Hemiptera) | Syria, Al Kseabiia – BSh/BSk/Csa |
| 55 | EABb 92/11-Dm | *D. maroccanus* (Acrididae, Acridoidea, Orthoptera) | Spain "La Serena"Badajoz – BSk |
| 56 | IMI 391362 | *E. integriceps* (Pentatomoidea, Hemiptera) | Syria – BSh/BSk/Csa |
| 57 | IMI 386700 | *Prostephanus truncatus* (Bostrichidae, Bostrichoidea, Coleoptera) | Kenya (Kibwezi) – Aw/BSh |
| 58 | Naturalis | Active compound: ATCC74040 (boll weevil cadaver) | USA, Texas, Rio Grande Valley – Cfa |
| 59 | 4044 PL | *Auchenorrhynchus* sp. (Heteroptera, Hemiptera) | Poland, Jeziory – Dfb |
| 60 | EABb 00/26-Su | Soil | Spain "la Serena" Badajoz – BSk |
| 61 | IMI 012943 | *Scolytus intricata* (Curculionidae, Curculionoidea, Coleoptera) | UK – Cfb |
| 62 | IMI 358840 | *Otiorhynchus sulcatus* (Curculionidae, Curculionoidea) | UK, England – Cfb |
| 63 | IMI 391043 | *E.* sp. (Pentatomoidea, Hemiptera) | Syria – BSh/BSk/Csa |
| 64 | IMI 391361 | *E. integriceps* (Pentatomoidea, Hemiptera) | Syria – BSh/BSk/Csa |
| 65 | IMI 393156 | *Panolis flammea* (Noctuidae, Noctuoidea, Lepidoptera) | United Kingdom – Cfb |
| 66 | IMI 393157 | *Panolis flammea* (Noctuidae, Noctuoidea, Lepidoptera) | ? |
| 67 | SP IR 500 | *E. integriceps* (Pentatomoidea, Hemiptera) | Iran – BSk or Csa |
| 68 | SP KR 403 | *E. integriceps* (Pentatomoidea, Hemiptera) | Kyrgyz Republ. – Dsa |
| 69 | SP KZ 467 | *E. integriceps* (Pentatomoidea, Hemiptera) | Khazakstan – Dsa |
| 70 | SP R 134 -  ARSEF 6008 | *E. integriceps* (Pentatomoidea, Hemiptera) | Russia, Volgograd – BSk |
| 71 | SP R159 -  ARSEF 6011 | *E. integriceps* (Pentatomoidea, Hemiptera) | Russia, Krasnii Sulin, Rostov – Dfa/Dsa |
| 72 | SP R 171 -  ARSEF 6015 | *E. integriceps* (Pentatomoidea, Hemiptera) | Russia, Krasnii Sulin, Rostov – Dfa/Dsa |
| 73 | SP R 184 -  ARSEF 6019 | *E. integriceps* (Pentatomoidea, Hemiptera) | Russia, Volgograd – BSk |
| 74 | SP IR 582 | *E. integriceps* (Pentatomoidea, Hemiptera) | Iran – BSk or Csa |
| 75 | SP O46 | *E. integriceps* (Pentatomoidea, Hemiptera) | Turkey – BSk or Csa or Dsa |
| 76 | SP U 259 -  ARSEF 6060 | *E. integriceps* (Pentatomoidea, Hemiptera) | Uzbekistan Southwest of Samarkand, Sazagan – BSk or Dsa/Dsb |
|  | ***B. brongniartii*** |  |  |
| 77 | IMBST 95031 | *Melolonta melolonta* (Coleoptera: Scaraboeoidae) | Switzerland |
| 78 | 1724 | - | - |
| 79 | 291 | *Melolonta hippocastani* (Coleoptera: Scaraboeoidae) | Germany |
|  | ***Beauveria* species** |  |  |
| 80 | *B. cylindrosporum* 1727 | Not specified | Not specified |
| 81 | *B. felina* IMI 159339  (*Isaria felina*) | faecal pellets of *Phalanges* *maculatus* | Papua Nea Guinea |
| 82 | *B. felina* IMI 021166 (*Isaria felina*) | infected yeast | UK |
| 83 | *B. felina* IMI 077369  (*Isaria felina*) | *Theobroma* cacao beans | Ghana |
| 84 | *B. geodes* ARSEF 2684 (*Tolypocladium geodes*) | Not specified | Not specified |
| 85 | *B. nivea*  ARSEF 4888 (*T. inflatum*) | Larva *(*Coleoptera: Scarabaeidae, Aphodiinae**)** | USA, Michigan Hollow State Forest, New York |
| 86 | *B. nivea* 1552 | - | - |
| 87 | *B. nubicola* ARSEF 3434 (*T. nubicola*) | Soil | Canada, Mt Allen, Kananaskis Valley, Alberta |
| 88 | *B. parasitica* ARSEF 3436  (*T. parasiticum*) | Bdelloid rotifers in farm soils | Canada, Guelph, Ontario |
| 89 | *B. sulfurescens* 1719 | Non - identified | France |
| 90 | *B. tundrensis* ARSEF 3400  (*T. tundrense*) | Soil | Canada, Devon Island, Northwest Territories |
| 91 | *B. vermiconia* 1717 | Soil (Volcanic ash) | Chile |
| 92 | *B. vermiconia*  IMI 320027 | volcanic ash soil | Chile |
| 93 | *B. vermiconia* IMI 342563 (CBS 849.73) | volcanic ash | Chile |
|  | **Other Hypocreales sp** |  |  |
| 94 | *Hirsutella longicolla*  1458 | - | - |
| 95 | *H. subulata* 1457 | **-** | - |
| 96 | *Paecilomyces fumosoroseus* 11PFR | ***Melolontha melolontha*** or *Carpocapsa pomonella* (codling moth = **Lepidoptera**) | Switzerland or Austria |
| 97 | *P. lilacinus* ARSEF 2182 | Egg mass *Meloidogyne* sp. (Tylenchida: Heteroderidae) on potato | Philippines, Ormoc, Leyte |
| 98 | *Nomurea rileyi*  ARSEF 6881 | *Platypena scabra* (Noctuidae, Lepidoptera) | USA, Quincy, Florida |
| 99 | *Aschershonia* 2268 | *Trialeurodes vaporariorum* (Aleurodidae, Homoptera) | Utrecht, Netherlands |
| 100 | *Cordyceps militaris* 0110 | **-** | - |
| 101 | *Tolypocladium* sp.  1740 | **-** | - |
| 102 | *Pochonia chlamydosporia* IMI 113169 | Soil | Canada |
| 103 | *Haptocillium balanoides* ARSEF 3350 | Nematodes: Secernentea | USA, California |
| 104 | *Simplicillium lamellicola* 234410 | *Azolla* sp. | UK |

-: no information available

*ATHUM, University of Athens Fungal Collection, Athens Greece; ARSEF, US Department of Agriculture, Agricultural Research Service Collection of Entomopathogenic Fungal Cultures; IMI, Genetic Resource Collection at CABI Bioscience, Egham, UK; CBS, Centraalbureau voor Schimmelcultures, Utrecht, The Netherlands; Bb and isolates with single numbers were kindly provided by Dr. A. Vey and Dr. Y. Couteaudier (INRA, France), isolates EABb by Dr. E. Quesada-Moraga (University of Cordoba, Spain), isolates Ht1 and Fo1 by Dr. E. Beerling (Applied Plant Research, Division Glasshouse Horticulture, Wageningen, The Netherlands), isolates SP by Dr. D. Moore (CABI, UK) and isolates PL by Dr. C. Tkacszuk (Poland).

** Af, Tropical Rain Forest; Am, Tropical Monsoon climate; Aw, Tropical wet and dry; BWh, Dry (arid and semiarid) desert low latitude climate; BWk, Dry (arid and semiarid) desert middle latitude climate; BSh, Dry (arid and semiarid) steppe low latitude climate; BSk, Dry (arid and semiarid) steppe middle latitude climate; Csa/Csb, Temperate Mediterranean climate; Cfa/Cwa, Temperate humid subtropical climate; Cfb/Cwb/Cfc, Temperate Maritime climate; Cwb, Temperate with dry winters climate; Cfc, Temperate Maritime Subarctic climate; Dfa/Dwa/Dsa, Hot summer Continental climate; Dfb/Dwb/Dsb, Warm summer Continental climate; Dfc/Dwc/Dsc, Continental Subarctic climate; Dfd/Dwd, Continental Subarctic climate with extrememly severe winters [41].
