# Supplementary material for: Phylogenetic and biogeographic implications inferred by mitochondrial intergenic region analyses and ITS1-5.8S-ITS2 of the entomopathogenic fungi Beauveria bassiana and B. brongniartii
Source: BMC Microbiol. 2010 Jun 16;10:174. doi: 10.1186/1471-2180-10-174 (PMC2896372; doi:10.1186/1471-2180-10-174)
Supplement: Additional File 3 — PCR amplicon sizes (in nucleotides) of all B. bassiana isolates studied for the mt intergenic regions nad3-atp9 and atp6-rns. ITS1-5.8S-ITS2 amplicons are not shown because they were more or less identical (ranging from 480-482 nt for all strains). [file 1471-2180-10-174-S3.DOC]

**Additional File 3, Table S3** - **PCR amplicon sizes (in nucleotides) of all *B. bassiana* isolates studied for the mt intergenic regions *nad*3-*atp*9 and *atp*6-*rns*.** ITS1-5.8S-ITS2 amplicons are not shown because they were more or less identical (ranging from 480-482 nt for all strains).

|  | **Host** | | ***nad*3-*atp*9** | ***atp*6-*rns*** | **Climate*** | **Group/**  **Sub-group** |
| --- | --- | --- | --- | --- | --- | --- |
| **Strain** | **Genus** | **Order** | **Intergenic region** | **Intergenic region** |  |  |
| ***B. bassiana*** |  |  |  |  |  |  |
| **Clade A** |  |  |  |  |  |  |
| Bb147 | *Ostrinia* | Lepidoptera | 302 | 439 | Cfb | A/1 |
| Bb170 | *Ostrinia* | Lepidoptera | 302 | 439 | Dwb | A/1 |
| Bb307 | *Ostrinia* | Lepidoptera | 302 | 439 | Cfb? | A/1 |
| Bb338 | *Ostrinia* | Lepidoptera | 302 | 439 | Cfb | A/1 |
| Bb512 | *Ostrinia* | Lepidoptera | 302 | 440 | Cfb? | A/1 |
| BbFo1 | *Frankliniella* | Thysanoptera | 302 | 440 |  | A/2 |
| IMI298057 | *Pantorhytes* | Coleoptera | 301 | 443 | Af | A/2 |
| IMI298058 | *Pseudodoniella* | Hemiptera | 300 | 440 | Af | A/2 |
| IMI 331274 | *Grasshopper* | Orthoptera | 301 | 428 | Cfa | A/2 |
| IMI 348041 | *Emmalocera* | Lepidoptera | 302 | 440 | Cfa | A/2 |
| EABb 01/33-Su | Soil |  | 301 | 457 | Csa | A/3 |
| EABb 06/02-Hy | *Hyalopterus* | Hemiptera | 302 | 444 | ? | A/3 |
| EABb 06/03-Ct | *Capnodis* | Coleoptera | 332 | 446 | ? | A/3 |
| EABb 91/6-Ci | *Calliptamus* | Orthoptera | 301 | 454 | ? | A/3 |
| EABb 91/7-Dm | *Dociostaurus* | Orthoptera | 301 | 455 | Bsk | A/3 |
| EABb 93/14-Tp | *Thaumatopoea* | Lepidoptera | 298 | 458 | Csa | A/3 |
| Ht1 | *Hoplothrips* | Thysanoptera | 301 | 442 | ? | A/3 |
| SP 3 372 | *Eyrygaster* | Hemiptera | 301 | 456 | Csa/Bsh/Bsk | A/3 |
| EABb 01/12-Su | Soil |  | 302 | 442 | Csa | A/4 |
| EABb 01/88-Su | Soil |  | 303 | 445 | Csa | A/4 |
| EABb 01/103-Su | Soil |  | 302 | 447 | Csa | A/4 |
| ATHUM 4946 | Air |  | 302 | 440 | Csa | A/5 |
| EABG 00/23-Su | Soil |  | 302 | 440 | Bwk | A/5 |
| EABb 04/01-Tip | *Timaspis* | Hymenoptera | 302 | 440 | Csa | A/5 |
| IMI 344464 | *Ctenarytaina* | Hemiptera | 302 | 440 | Csb | A/5 |
| IMI 391044 | *Eyrygaster* | Hemiptera | 302 | 440 | Csa/Bsh/Bsk | A/5 |
| SP 2 268 | *Eyrygaster* | Hemiptera | 303 | 439 | Csa/Bsh/Bsk | A/5 |
| SP 2 273 | *Eyrygaster* | Hemiptera | 313 | 439 | Csa/Bsh/Bsk | A/5 |
| SP 2 315 | *Eyrygaster* | Hemiptera | 302 | 439 | Csa/Bsh/Bsk | A/5 |
| SP 2 321/1 | *Eyrygaster* | Hemiptera | 302 | 440 | Csa/Bsh/Bsk | A/5 |
| B21 | *Auchenorrynchus* | Hemiptera | 260 | 428 | Dfb | A/6 |
| B22 | “spider” | Arachnida | 260 | 428 | Dfb | A/6 |
| Bb169 | *Sitona* | Coleoptera | 260 | 428 | Cfb | A/6 |
| Bb216 | *Sitona* | Coleoptera | 260 | 428 | Csa/Bsk/Bsh | A/6 |
| Bb220 | *Sitona* | Coleoptera | 260 | 428 | Cfb | A/6 |
| Bb228 | *Sitona* | Coleoptera | 260 | 426 | Cfb | A/6 |
| Bb327 | *Sitona* | Coleoptera | 260 | 426 | Cfb | A/6 |
| IMI 386694 | *Popillia* | Coleoptera | 260 | 427 | Csa/Csb | A/6 |
| 4157/1 | *Anthocoris* | Hemiptera | 260 | 428 | Cfb | A/6 |
| 4157/4 | “spider” | Arachnida | 260 | 428 | Cfb | A/6 |
| 4157/5 | *Anthocoris* | Hemiptera | 260 | 428 | Cfb | A/6 |
| 4157/6 | *Ichneumonidae* | Hymenoptera | 260 | 428 | Cfb | A/6 |
| IMI 044229 | *Diatrea* | Lepidoptera | 260 | 436 | Am/Aw | A/7 |
| IMI 331267 | *Hypothenemus* | Coleoptera | 302 | 434 | Cfa (Cwa) | A/7 |
| IMI 386696 | *Diabrotica* | Coleoptera | 259 | 440 | Af | A/7 |
| IMI 392611 | “spider” | Arachnida | 260 | 432 | Af | A/7 |
| IMI 393155 | *Phlebotomus* | Diptera | 260 | 436 | Cfb | A7 |
| IMI 331266 | Acrididae | Orthoptera | 259 | 441 | BSh | A |
| IMI 386705 | *Diabrotica* | Coleoptera | 262 | 439 | BSh | A |
| IMI392612 | “spider” | arachnida | 302 | 439 | Af | A |
| IMI 391363 | *Eyrygaster* | Hemiptera | 302 | 439 | Csa/Bsh/Bsk | A |
| IMI 391704 | *Eyrygaster* | Hemiptera | 302 | 439 | Csa/Bsh/Bsk | A |
| IMI 348083 | *Phoracantha* | Coleoptera | 274 | 439 | Csa | A |
| IMI 331273 | *Tenebrinoid* | Coleoptera | 302 | 440 | Aw/Bsh | A |
| EABb 92/11-Dm | *Dociostaurus* | Orthoptera | 301 | 455 | Bsk | A |
| IMI 391362 | *Eyrygaster* | Hemiptera | 305 | 422 | Csa/Bsh/Bsk | A |
| Naturalis | Commercial |  |  | ND | Csa | A |
| IMI 386700 | *Prostephanus* | Coleoptera | 302 | 443 | Aw/BSh | A |
| **Clade C** |  |  |  |  |  |  |
| 4044 | *Auchenorrhynchus* | Hemiptera | 275 | 451 | Dfb | C |
| EABG 00/26-Su | Soil |  | 276 | 455 | Bsk | C |
| IMI 012943 | *Scolytus* | Coleoptera | 275 | 451 | Cfb | C |
| IMI 358840 | *Otiorhynchus* | Coleoptera | 274 | 450 | Cfb | C |
| IMI 391043 | *Eyrygaster* | Hemiptera | 276 | 468 | Csa/Bsh/Bsk | C |
| IMI 391361 | *Eyrygaster* | Hemiptera | 275 | 451 | Csa/Bsh/Bsk | C |
| IMI 393156 | *Panolis* | Lepidoptera | 277 | 442 | Cfb | C |
| IMI 393157 | *Panolis* | Lepidoptera | 277 | 442 | ? | C |
| SP IR500 | *Eyrygaster* | Hemiptera | 277 | 453 | Bsk/Csa | C |
| SPKR403 | *Eyrygaster* | Hemiptera | 275 | 450 | Dsa | C |
| SP KZ467 | *Eyrygaster* | Hemiptera | 275 | 283 | Dsa | C |
| SP R134 | *Eyrygaster* | Hemiptera | 275 | 451 | Bsk | C |
| SP R159 | *Eyrygaster* | Hemiptera | 275 | 449 | Dfa/Dsa | C |
| SP R171 | *Eyrygaster* | Hemiptera | 275 | 450 | Dfa/Dsa | C |
| SP R184 | *Eyrygaster* | Hemiptera | 274 | 483 | Bsk | C |
| **Clade A_2_** |  |  |  |  |  |  |
| SP IR582 | *Eyrygaster* | Hemiptera | 263 | 426 | Bsk/Csa | A_2_ |
| SP O46 | *Eyrygaster* | Hemiptera | 262 | 426 | Bsk/Csa/Dsa | A_2_ |
| SP U259 | *Eyrygaster* | Hemiptera | 263 | 428 | Bsk/Dsa/Dsb | A_2_ |
| ***B. brongniartii*** |  |  |  |  |  |  |
| IMBST 95031 | *Melolontha* | Coleoptera | 289 | 526 |  |  |
| Bbr 1724 | - | - | 289 | 525 |  |  |
| IMBST 291 | *Melolontha* | Coleoptera | 289 | 525 |  |  |

ND: Not detected

* Af, Tropical Rain Forest; Am, Tropical Monsoon climate; Aw, Tropical wet and dry; BWh, Dry (arid and semiarid) desert low latitude climate; BWk, Dry (arid and semiarid) desert middle latitude climate; BSh, Dry (arid and semiarid) steppe low latitude climate; BSk, Dry (arid and semiarid) steppe middle latitude climate; Csa/Csb, Temperate Mediterranean climate; Cfa/Cwa, Temperate humid subtropical climate; Cfb/Cwb/Cfc, Temperate Maritime climate; Cwb, Temperate with dry winters climate; Cfc, Temperate Maritime Subarctic climate; Dfa/Dwa/Dsa, Hot summer Continental climate; Dfb/Dwb/Dsb, Warm summer Continental climate; Dfc/Dwc/Dsc, Continental Subarctic climate; Dfd/Dwd, Continental Subarctic climate with extrememly severe winters (36).
