# Supplementary material for: Phylogenetic and biogeographic implications inferred by mitochondrial intergenic region analyses and ITS1-5.8S-ITS2 of the entomopathogenic fungi Beauveria bassiana and B. brongniartii
Source: BMC Microbiol. 2010 Jun 16;10:174. doi: 10.1186/1471-2180-10-174 (PMC2896372; doi:10.1186/1471-2180-10-174)
Supplement: Additional File 4 — Values of symmetric difference between the phylogenetic trees produced from ITS1-5.8S-ITS2, nad3-atp9, atp6-rns and the concatenated dataset with NJ, BI and MP methods. [file 1471-2180-10-174-S4.DOC]

**Additional File 4, Table S4 –** **Values of symmetric difference between the phylogenetic trees produced from ITS1-5.8S-ITS2, *nad*3-*atp*9, *atp*6-*rns* and the concatenated dataset with NJ, BI and MP methods.**

|  |  | **ITS1-5.8S-ITS2** | | | **nad3-atp9 (N3-A9)** | | | **atp6-rns (A6-R)** | | | **Concatenated (Con)** | | |
| --- | --- | --- | --- | --- | --- | --- | --- | --- | --- | --- | --- | --- | --- |
|  |  | NJ | BI | MP* | NJ | BI | MP* | NJ | BI | MP* | NJ | BI | MP* |
| **ITS** | NJ | 0 | 128 | 126 |  |  |  |  |  |  |  |  |  |
|  | BI | 128 | 0 | 14 |  |  |  |  |  |  |  |  |  |
|  | MP* | 126 | 14 | 0 |  |  |  |  |  |  |  |  |  |
| **N3-A9** | NJ |  |  |  | 0 | 87 | 85 |  |  |  |  |  |  |
|  | BI |  |  |  | 87 | 0 | 24 |  |  |  |  |  |  |
|  | MP* |  |  |  | 85 | 24 | 0 |  |  |  |  |  |  |
| **A6-R** | NJ |  |  |  |  |  |  | 0 | 69 | 67 |  |  |  |
|  | BI |  |  |  |  |  |  | 69 | 0 | 24 |  |  |  |
|  | MP* |  |  |  |  |  |  | 67 | 24 | 0 |  |  |  |
| **Con** | NJ |  |  |  |  |  |  |  |  |  | 0 | 86 | 86 |
|  | BI |  |  |  |  |  |  |  |  |  | 86 | 0 | 40 |
|  | MP* |  |  |  |  |  |  |  |  |  | 86 | 40 | 0 |

*: In this analysis, the 50% majority-rule consensus tree produced of the specific dataset from all the parsimonious trees was used.
