# Supplementary material for: Phylogenetic and biogeographic implications inferred by mitochondrial intergenic region analyses and ITS1-5.8S-ITS2 of the entomopathogenic fungi Beauveria bassiana and B. brongniartii
Source: BMC Microbiol. 2010 Jun 16;10:174. doi: 10.1186/1471-2180-10-174 (PMC2896372; doi:10.1186/1471-2180-10-174)
Supplement: Additional File 7 — PCR primer pairs used for the amplification of the complete mt genomes of B. bassiana Bb 147 and B. brongniartii IMBST 95031 and approximate amplicon sizes in bp. [file 1471-2180-10-174-S7.DOC]

**Additional File 7, Table S7 - PCR primer pairs used for the amplification of the complete mt genomes of *B. bassiana* Bb 147 and *B. brongniartii*** IMBST 95031 and approximate amplicon sizes in bp.

| **Primer Pairs** | ***B. bassiana***  **amplicons (bp)** | ***B. brongniartii***  **amplicons (bp)** |
| --- | --- | --- |
| ssuF/lsusR | 4600 | 5000 |
| lsusF/lsueR | 1500 | 2900 |
| lsusF/nad3R | 8800 | 8800 |
| nad3F/nad5R | 3800 | 3200 |
| nad5F/cobR | 3000 | 3500 |
| cobF/cox1R | 3200 | 3600 |
| cox1F/nad1R | 4200 | 2400 |
| nad1F/ssuR | 4100 | 4200 |
